# Online optimization of continuous casting cutting

**DOI:** 10.1038/s41598-025-08908-0

**Published:** 2025-07-04

**Authors:** Jing Li, Jianjing Zhang, Xiaoru Xing, Sixiao Hu, Yixuan Shi

**Affiliations:** Hebei Institute of Mechanical and Electrical Technology, Xingtai, 054000 Hebei China

**Keywords:** Continuous casting, Online optimization, Nested optimization, Real-time decision making, Steel production efficiency, Mathematics and computing, Applied mathematics

## Abstract

Continuous casting is a key process in steel production, and optimizing the cutting process to minimize material waste while meeting customer demands is one of the core challenges in improving production efficiency. This paper proposes an online optimization algorithm to address the cutting optimization problem in steel continuous casting. Through an optimization model constructed by combining nested models, analytical modeling, and computer traversal algorithms, cutting schemes can be adjusted in real-time when production abnormalities occur, ensuring production continuity and efficiency. The proposed method demonstrates excellent performance in reducing cutting losses, meeting production requirements, and minimizing secondary cutting operations, with broad practical application potential.

**Supplementary Information:**

The online version contains supplementary material available at 10.1038/s41598-025-08908-0.

## Introduction

### Research background

**Fig. 1:**
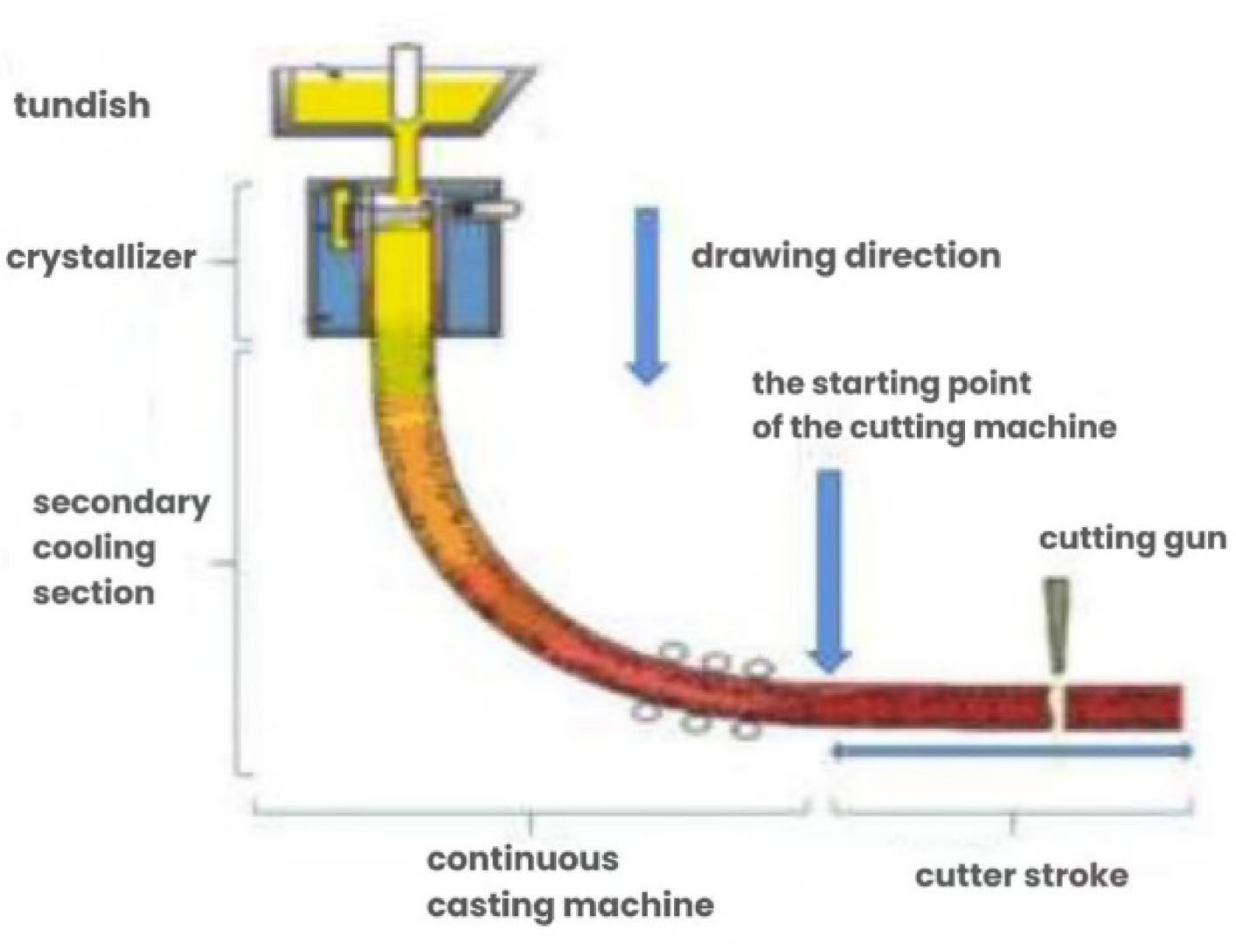
Schematic Diagram of the continuous casting process.

**Fig. 2:**
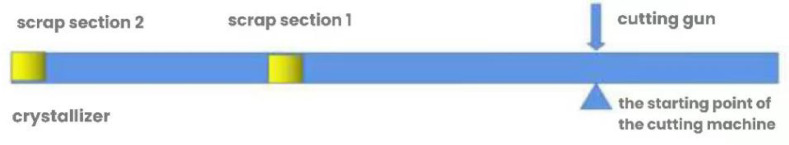
Schematic diagram of the scrap section (abnormal segment).

Continuous casting is a crucial technological process in steel production, involving the continuous transformation of molten steel into solid steel billets. The general process of continuous casting is illustrated in Fig. 1. In this process, billet cutting is a critical step that not only determines the final shape and dimensions of products but also directly affects material utilization and production efficiency^1^. To improve economic benefits, steel plants need to minimize material waste while optimizing cutting processes under the premise of meeting customer requirements. However, production processes inevitably encounter issues such as crystallizer abnormalities and billet quality fluctuations, which may lead to increased material losses and even affect production continuity^2^. These abnormalities can result in the formation of scrap sections along the billet, as illustrated in Fig. 2.

Traditional cutting optimization methods primarily focus on static optimization, designing optimal cutting schemes for predetermined billet lengths. These methods assume stable production conditions without any emergencies. However, in actual production, billet cutting not only needs to ensure production continuity but must also dynamically respond to various abnormal situations that occur during production, such as equipment failures or billet defects^3^. In these situations, static optimization methods often prove ineffective, usually requiring manual intervention or temporary adjustment of cutting schemes, resulting in decreased production efficiency and increased material waste^4^. Therefore, developing algorithms capable of real-time optimization of cutting schemes in dynamic production environments has become a key issue for improving automation levels and production efficiency in the steel manufacturing industry^5^.

### Problem context and research objectives

To promote in-depth research on continuous casting cutting optimization, the 2021 "Higher Education Press Cup" China Undergraduate Mathematical Contest in Modeling^6^ presented a problem based on actual industrial background, requiring participants to solve optimization problems in continuous casting cutting. Solving this competition problem provides theoretical support and algorithmic reference for cutting optimization in actual production.

Based on this competition problem, this study aims to propose an online optimization algorithm capable of addressing various complex issues in actual continuous casting cutting processes. Although the competition problem simplifies actual issues, it still represents typical challenges of this practical problem and provides a good platform for developing new algorithms. The competition problem mainly includes two aspects of optimization: first, the static optimization problem—determining optimal cutting schemes for a series of given billet tail lengths; second, the online optimization problem—real-time adjustment of cutting schemes when abnormalities occur in the crystallizer to ensure production continuity and efficiency. Solving these problems has both theoretical significance and provides important reference for sustainable development in actual industrial production. Despite receiving thousands of submissions during the competition, due to the complexity of the problem, no effective solutions were provided for the dynamic optimization aspect of the problem. Therefore, further post-competition research is necessary to explore more advanced optimization methods.

### Gaps and challenges in existing research

Although existing research has made progress in continuous casting cutting optimization, most methods primarily focus on static optimization and still face significant challenges in addressing real-time abnormal requirements during production^4,5^. When crystallizer abnormalities, billet quality issues, or other emergencies occur, existing methods often rely on manual intervention or manual adjustments, failing to achieve fully automated production processes.

One of the most authoritative studies in this field is the post-competition research conducted by the expert review committee of the competition organizers, including the works of Xue^7^ and Cai^8^. These studies were published in the official journal of the competition committee and represent the most influential models and methodologies proposed for this problem, demonstrating excellent performance in static optimization. However, despite their significant contributions, they exhibit poor adaptability in handling dynamic abnormal adjustments and lack real-time automated processing capability.

Existing online optimization algorithms struggle to ensure a globally optimal cutting strategy, and this issue becomes even more pronounced when abnormal segments occur frequently or at short intervals. Some approaches adopt a strategy of dividing the billet into multiple sub-intervals for separate local optimization. However, due to the lack of a global perspective, such methods often lead to increased cutting losses or production line disruptions when dealing with complex abnormal conditions. At present, there is still a lack of an efficient algorithm capable of dynamically adjusting the cutting strategy in real time under a dynamic environment. Therefore, developing a novel online optimization algorithm is of great theoretical and practical significance for improving production efficiency and reducing material loss.

### Key innovations and contributions

This study proposes an innovative online optimization algorithm aimed at solving cutting optimization problems in steel continuous casting. Specifically, our algorithm demonstrates significant innovation and superiority in both static and online optimization aspects.

First, in static optimization problems, our algorithm significantly outperforms traditional integer programming methods in computational speed. For example, compared to methods proposed by Wang^4^ and Hu^5^, our algorithm is several orders of magnitude faster. This significant speed improvement not only enhances static optimization efficiency but also lays the foundation for real-time online optimization algorithms. Through optimizing computational processes and algorithm structures, we achieve efficient calculation with large-scale data, ensuring feasibility in actual production applications.

Second, in online optimization problems, we construct a novel optimization model by combining nested multi-layer optimization, analytical modeling, and computer enumeration methods, successfully addressing all shortcomings in existing research. Specifically, our model can adjust cutting schemes in real-time when abnormalities occur during production, ensuring production continuity and efficiency. This method not only reduces cutting losses and meets production requirements but also minimizes secondary cutting operations, demonstrating excellent performance. During dynamic adjustments, the model can quickly respond to abnormal situations and automatically generate new cutting plans, greatly improving production line flexibility and reliability.

Furthermore, we developed multiple automated software tools for verifying and demonstrating algorithm accuracy and automation feasibility. These tools not only automatically calculate cutting schemes but also intuitively demonstrate the online optimization process through dynamic demonstration software. As verification and demonstration tools, these applications prove the algorithm’s feasibility and effectiveness in actual production environments. In the future, after further modification and integration, the optimization algorithm will be able to connect directly with production equipment through programming interfaces or control systems, achieving fully automated cutting operations without human intervention.

### Research approach and methodological contributions

In the field of modern industrial optimization, traditional mathematical models such as integer programming and heuristic algorithms often struggle to directly solve complex multi-objective optimization problems. Therefore, conducting analytical exploration for specific problems and integrating parameterized optimization methods has become an essential approach to improving computational efficiency. This study proposes a novel mathematical optimization approach based on a nested optimization framework. By employing multi-layer modeling and parameterized enumeration techniques, the proposed method enables efficient solutions to complex industrial problems.

Existing research has demonstrated the significant value of nested optimization methods in multi-objective and cross-domain engineering optimization problems. For example, Evins^9^ applied a multi-level optimization method to integrate building design, energy system design, and operation scheduling, optimizing overall energy efficiency. Similarly, Fathy et al.^10^ achieved coordinated optimization of passive and active control in automotive suspension systems.

Likewise, parameterized optimization methods have been widely studied in discrete optimization problems.Hara and Ishihata^11^ explored the theoretical foundations of parameterized enumeration algorithms, while Creignou et al.^12^ provided key references for the parameter-solving strategies in this study.

In industrial optimization, nested optimization methods have been successfully applied to steel cutting optimization^1^ and parameter optimization in complex system design^13^. Additionally, in dynamic optimization problems, Dörterler et al.^14^ employed multi-objective nested optimization in robot gripper optimization, and Groppen and Berko^15^ introduced modular enumeration techniques in discrete optimization. These studies indicate that nested optimization demonstrates strong adaptability and robustness in dynamic environments.

Building upon these studies, this research extends the approach by proposing a four-layer nested optimization model and conducting an in-depth analysis of optimization problems at each level. By using the optimization results from each layer as inputs for higher-level optimization, this framework forms a multi-module, multi-layered optimization structure, making it a powerful tool for solving complex real-world problems. Compared to the local tuning strategy proposed by Gergel et al.^16^, this study integrates nested structures with parameterized enumeration, further enhancing optimization efficiency and solution accuracy.

### Paper structure

The remainder of this paper is organized as follows:

- Section "Model establishment and solution" presents the problem definition, optimization modeling, and solutions for static and online optimization problems.
- Section "Discussion" evaluates the feasibility of the proposed model in real-world production, discusses its adaptability to different production requirements, and highlights its scalability and potential for industrial implementation.
- Section "Conclusion" summarizes the contributions of this study and outlines future research directions and industrial applications.

## Model establishment and solution

### Competition problem definition and optimization problem definition

#### Problem description and requirements

The competition problem includes two aspects of optimization requirements: (1) Static optimization problem, i.e., determining the optimal cutting scheme given a series of billet tail lengths; (2) Online optimization problem, i.e., real-time adjustment of cutting schemes when abnormalities occur in the crystallizer to ensure production continuity and efficiency.

Under conditions satisfying process parameters, basic requirements, and normal requirements, the competition problem mainly includes two specific issues:

1. *Static optimization problem*: Provide the optimal cutting scheme for a series of specific billet tail lengths.
2. *Online optimization problem*: Provide real-time optimal cutting schemes when abnormalities occur in the crystallizer.

We refined the requirements in the problem. First, the cutting length must be between 4.8 m and 12.6 m, otherwise it cannot be transported away, obstructing production, which is unacceptable and can be considered as a Level 0 optimization objective.

Secondly, users will propose their target requirements, including the upper limit, lower limit, and target value of the required steel billet length. Due to production process limitations, the lower limit must be greater than or equal to 8.0 m, the upper limit must be less than or equal to 11.6 m, and the target value is between the upper and lower limits. If the cut length is less than the user’s required lower limit, the entire steel billet is scrapped, generating cutting loss. If the cut length is greater than the user’s required upper limit, it is transported away for secondary offline cutting, and the cut-off part is scrapped as cutting loss. Minimizing cutting loss is the Level 1 optimization objective.

Next, maximizing the number of steel billets equal to the user’s target value is the Level 2 optimization objective.

Finally, minimizing the number of offline cuttings is the Level 3 optimization objective.

Other process descriptions, such as the steel billet moving at 1 m per minute, the cutting machine taking 3 min for one cut, and 1 min to return to the cutting starting point, essentially require that the cutting length cannot be less than 4 m, which is already included in the constraint that the steel billet length cannot be less than 4.8 m, so there is no need to consider this constraint separately. This is also a widely acknowledged consensus in existing research.

#### Mathematical expression of existing research limitations

**Fig. 3:**
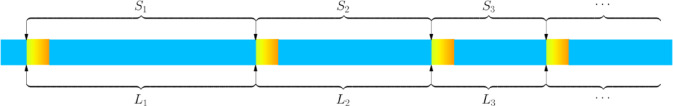
Schematic diagram of coordinate intervals between abnormal segments and sub-intervals.

The specific deficiencies of existing best models, represented by the official expert committee of the competition organizers’ post-competition research^7,8^, are reflected in:

As shown in Fig. 3, ${S}_{i}$ represents the coordinate interval between two abnormal segments, and ${L}_{i}$ represents the sub-interval.

From the perspective of meeting transportation conditions, when at least one interval ${S}_{i}$ is less than 4.8 m, existing solutions cannot automatically find a solution that satisfies the transportation conditions.

From the perspective of minimizing cutting loss, we set the user’s target lower limit as ${Y}_{1}$. In most cases, existing solutions can find the solution with the minimum overall cutting loss, but when at least one interval ${S}_{i}\in (12.6K,{KY}_{1}+4.8),K<\frac{4.8}{12.6-{Y}_{1}},K\in {Z}^{+}$ appears, the solution obtained by existing methods will have significant deviations from the solution with the minimum overall cutting loss. For example: If the user’s target lower limit ${Y}_{1}=9.0$ meters, and there exists ${S}_{i}=13.7$ meters, dividing ${L}_{i}$ according to ${L}_{i}{=S}_{i}=13.7$ meters, the cutting loss of the ${L}_{i}$ interval will reach 13.7 m. In reality, if the division boundary is moved slightly, making ${L}_{i}=13.8$ meters, the cutting loss of the ${L}_{i}$ interval will be significantly reduced to 4.8 m.

From the perspective of maximizing user satisfaction, under the premise of minimizing cutting loss, for a small number of cases, the solution obtained by existing methods deviates from the globally optimal solution in terms of user satisfaction. For example: If the user’s target value ${Y}_{0}=9.5$ meters, the user’s target lower limit ${Y}_{1}=9.0$ meters, the user’s target upper limit ${Y}_{2}=10.0$ meters, and there exists ${S}_{i}=14.2$ meters, ${S}_{i+1}=9.6$ meters, if divided according to ${L}_{i}{=S}_{i}=14.2$ meters, ${L}_{i+1}{=S}_{i+1}=9.6$ meters, the sum of the number of steel billets equal to the user’s target value in these two intervals is 1. If the boundary is moved to ${L}_{i}=14.3$ meters, ${L}_{i+1}=9.5$ meters, under the premise of maintaining minimum cutting loss, the sum of the number of steel billets equal to the user’s target value in these two intervals increases to 2.

From the perspective of minimizing the number of offline cuttings, existing solutions often fail to achieve the minimum number of offline cuttings. Some existing solutions reduce the number of offline cuttings through manual secondary adjustments, which does not meet the requirements of automated production.

These four major deficiencies highlight the significant limitations of existing methods in handling dynamic optimization problems in continuous casting cutting. Notably, Cai^8^ claims in his conclusion that his algorithm can obtain the optimal cutting scheme. However, a detailed analysis of his results and methodology indicates a clear inconsistency between this claim and the actual findings.

Although his paper mentions a manual adjustment in only one instance (modifying 4.7 m to 4.8 m), it does not systematically address the identified issues. At the same time, his result table still includes a 4.7 m cutting scheme, which violates the minimum transportable length constraint. Furthermore, while the second and third deficiencies do not explicitly appear in the example calculations, they remain potential risks in more general cases.

Additionally, the failure to minimize the number of secondary cuttings is also directly observable in his results, where some cutting schemes generate unnecessary secondary cutting operations without further optimization.

#### Expression explanation

1. In this paper, for concise expression, the length unit is no longer written, and the default unit for any number representing length is (meters). For example, 4.8 m will be directly written as 4.8.
2. In all schematic diagrams representing production status in this paper, the movement direction of steel billets in the figures is from left to right, which will not be marked in each figure.
3. In this paper, ${Y}_{0}$ represents the user’s target value, ${Y}_{1}(={Y}_{0}-0.5)$ represents the user’s target lower limit, and ${Y}_{2}(={Y}_{0}+0.5)$ represents the user’s target upper limit.

#### Optimization objectives

We establish three levels of optimization objectives:

- *First priority objective*: Minimize cutting loss.
- *Second priority objective*: Maximize the number of steel billets equal to the user’s target value.
- *Third priority objective*: Minimize the number of offline cuttings.

#### Model assumptions

Just like all the data provided in the problem for calculation, we set the user’s target value ${Y}_{0}$, the user’s target lower limit ${Y}_{1}$, and the user’s target upper limit ${Y}_{2}$ to be integer multiples of 0.1, and the occurrence time of abnormal scrap sections is also an integer multiple of 0.1 min. For situations where the occurrence time of abnormal segments is not an integer multiple of 0.1 min, we will discuss this at the end of the paper.

#### The multi-layer nested structure of the model

This model adopts a multi-layer nested structure, where each layer involves in-depth analysis and examination of the problem, and key parameter enumeration methods are applied where appropriate. The model is divided into four levels, as shown in Table 1:

**Table 1:** Hierarchical structure of the proposed model.

Level	Model content	Modeling method
1	Calculation of minimum cutting loss for a given number of usable billets (k)	Analytical modeling
2	Optimal cutting model for a steel billet with a fixed total length, also applicable to billet tail cutting	Analytical modeling, computational enumeration
3	Optimal cutting model for a steel billet with a fixed total length containing abnormal segments	Analytical modeling
4	Dynamic optimal cutting model	Analytical modeling, computational enumeration

Level 1 is a sub-model of Level 2, Level 2 is a sub-model of Level 3, and Level 3 is a sub-model of Level 4, forming a nested structure.

### Modeling and solving the static optimization problem (billet tail cutting)

Let the length of the billet tail to be cut be $L$, and the minimum cutting loss be $C(L)$. Among all possible cutting schemes, the billet tail may be cut into $k$ usable steel billets and $n$ individually scrapped steel billets during the online cutting process, where $k$ or $n$ may be greater than 0 or equal to 0. Some or all of the $k$ usable steel billets may need to undergo secondary offline cutting to meet user requirements. We set the total length of $k$ usable steel billets (before offline cutting) as ${L}_{k}$, and the total length of n individually scrapped steel billets as ${W}_{n}$. Obviously, ${L}_{k}+{W}_{n}=L$.

It is easy to know that the necessary condition for the billet tail to cut out k usable steel billets is $0 \le k \le \left\lfloor {\frac{L}{{Y_{1} }}} \right\rfloor$.

First, we establish a model to find the minimum cutting loss ${C}_{k}(k,L)$ based on any specified $k$ value under this premise.

#### Solving for the minimum cutting loss ${{{C}}}_{{{k}}}({{k}},{{L}})$ based on a specified ${{k}}$ value and its corresponding ${{{L}}}_{{{k}}}$ and ${{{W}}}_{{{n}}}$

We solve this in different cases. Case 1 and Case 2 may have an intersection, and we judge which case to adopt based on the superiority of the results in the intersection part. The following cases are all based on the premise of a specified $k$ value.

**Case 1:** $${{k}}{{{Y}}}_{1}\le {{L}}\le 12.6{{k}}$$

Case 1 is the necessary and sufficient condition for the existence of a cutting scheme that satisfies "${W}_{n}=0$ and meets transportation conditions". In this case, we solve for the minimum cutting loss ${C}_{k1}(k,L)$ based on ${W}_{n}=0$. ${C}_{k1}(k,L)$ is the minimum cutting loss solved based on the current values of $L$, $k$, and the premise of ${W}_{n}=0$.

**Sub-case 1.1:** $${{k}}{{{Y}}}_{1}\le {{L}}\le {{k}}{{{Y}}}_{2}$$

In this case, $L$ is directly cut into $k$ steel billets, resulting in ${C}_{k1}(k,L)=0$, ${L}_{k}=L$, ${W}_{n}=0$.

**Sub-case 1.2:** $${{k}}{{{Y}}}_{2}<{{L}}\le 12.6{{k}}$$

In this case, first cut $L$ into $k$ steel billets online, then perform secondary offline cutting on steel billets longer than ${Y}_{2}$ to ${Y}_{2}$ length, resulting in ${C}_{k1}(k,L)=L-k{Y}_{2}$, ${L}_{k}=L$, ${W}_{n}=0$.

**Case 2:** $${{L}}\ge {{k}}{{{Y}}}_{1}+4.8$$

Case 2 is the necessary and sufficient condition for the existence of a cutting scheme that satisfies "${W}_{n}>0$ and meets transportation conditions". In this case, we solve for the minimum cutting loss ${C}_{k2}(k,L)$ based on ${W}_{n}>0$. ${C}_{k2}(k,L)$ is the minimum cutting loss solved based on the current values of $L$, $k$, and the premise of ${W}_{n}>0$.

**Sub-case 2.1:** $${{k}}{{{Y}}}_{1}+4.8\le {{L}}<{{k}}{{{Y}}}_{2}+4.8$$

In this case, cut a single scrapped steel billet with a length of 4.8, then cut the remaining part into k usable steel billets, resulting in ${C}_{k2}(k,L)=4.8$, ${L}_{k}=L-4.8$, ${W}_{n}=4.8$.

**Sub-case 2.2:** $${{L}}\ge {{k}}{{{Y}}}_{2}+4.8$$

In this case, cut $k$ usable steel billets according to ${Y}_{2}$, and cut the remaining part into n individually scrapped steel billets, resulting in:

$${C}_{k2}(k,L)=L-k{Y}_{2}, {L}_{k}=k{Y}_{2}, {W}_{n}=L-k{Y}_{2}.$$

${C}_{k}(k,L)$ Is the minimum cutting loss based on the current $L$ and $k$. Cases 1 and 2 cover all situations of cutting schemes that satisfy transportation conditions based on the current $L$ and $k$. If $L$ and $k$ only conform to Case 1 or only conform to Case 2, calculate directly according to the corresponding case. However, since there may be an intersection between Case 1 and Case 2, if $L$ and $k$ conform to both Case 1 (based on ${W}_{n}=0$) and Case 2 (based on ${W}_{n}>0$), we calculate both and make a comparison. In this case:

$${C}_{k}(k,L)={\text{min}[C}_{k1}(k,L),{C}_{k2}(k,L)]$$

If ${C}_{k1}(k,L)={C}_{k2}(k,L)$, based on the principle of minimizing the number of secondary offline cuttings, we choose the scheme corresponding to ${C}_{k2}(k,L)$.

At this time, we have obtained the minimum cutting loss ${C}_{k}(k,L)$ based on the current $L$ and $k$, and the corresponding ${L}_{k}$ and ${W}_{n}$.

#### Traversing ${{k}}$ to solve for minimum cutting loss ${{C}}({{L}})$

$C(L)$ Is the minimum cutting loss for an billet tail of length $L$. After obtaining the result based on a specified $k$ value, we traverse all possible $k$ values to obtain the minimum cutting loss $C(L)$. If a certain $k$ does not have a cutting scheme that satisfies the transportation conditions for the current $L$, the program will automatically skip it during the traversal process. For example, when $L=13.6$, it will skip $k=1$.

$$0 \le k \le \left\lfloor {\frac{L}{{Y_{1} }}} \right\rfloor$$

$$C(L)=\text{min}[{C}_{k}(k,L)]$$

Through traversal, we can find the $k$ value corresponding to the minimum cutting loss. When $L<\lceil\frac{{{Y}_{1}Y}_{2}}{{Y}_{2}-{Y}_{1}}\rceil$, the k corresponding to the minimum cutting loss must be unique. When $L\ge \lceil\frac{{{Y}_{1}Y}_{2}}{{Y}_{2}-{Y}_{1}}\rceil$, there may be cases where the $k$ corresponding to the minimum cutting loss is not unique.

${D}_{1}({L}_{k},k)$ is the maximum number of steel billets equal to the user’s target value based on the current ${L}_{k}$ and $k$. If the $k$ value corresponding to the minimum cutting loss is not unique in the traversal results, we select the $k$ value with the maximum number of steel billets equal to the user’s target value ${D}_{1}({L}_{k},k)$, to meet the second priority objective. $D(L)$ is the maximum number of steel billets equal to the user’s target value that can be obtained for billet tail $L$ under the premise of minimizing cutting loss. The calculation method of ${D}_{1}({L}_{k},k)$ is the same as the calculation method of $D(L)$ in Section "Specific cutting scheme for the *L*_k_ Part". If ${L}_{k}$ and $k$ are the optimal values, then $D(L)={D}_{1}({L}_{k},k)$.

It should be especially noted that if during the traversal process, it is found that for any k value, there exists an L that conforms to sub-case 1.1, and $L<\lceil\frac{{{Y}_{1}Y}_{2}}{{Y}_{2}-{Y}_{1}}\rceil$, then we directly exit the loop, end the traversal, and return the result of sub-case 1.1. This is because At this point, the cutting loss is 0, and there is only one unique k value corresponding to the minimum cutting loss, which must be the optimal result. The advantage of doing this is that it can save computation time.

#### Specific cutting scheme for the ${{{L}}}_{{{k}}}$ part

Through traversal, we have found the optimal values of $k$, ${L}_{k}$, and ${L}_{n}$. The scheme to cut the ${L}_{k}$ part into $k$ steel billets may not be unique, and we need to find the cutting scheme that obtains the maximum number of steel billets equal to the user’s target value. For this, we provide online cutting schemes for different cases.

**Case 1:** $${{{k}}{{{Y}}}_{1}\le {{L}}}_{{{k}}}\le {{k}}{{{Y}}}_{0}$$

$$p = \left\lfloor {\frac{{L_{k} - kY_{1} }}{{Y_{0} - Y_{1} }}} \right\rfloor$$

$$q = \left\lfloor {\frac{{kY_{0} - L_{k} }}{{Y_{0} - Y_{1} }}} \right\rfloor$$

Cut $p$ steel billets according to ${Y}_{0}$, cut $q$ steel billets according to ${Y}_{1}$, cut $k-p-q$ steel billets according to ${L}_{k}-p{Y}_{0}-q{Y}_{1}$, $D(L)=p$.

For Case 1, we provide a proof that the above scheme is the cutting scheme with the maximum number of steel billets equal to the user’s target value, as follows:

First, prove that according to our scheme, p steel billets with length Y₀ can be cut out, and all cut steel billets conform to the user’s target range. We need to prove: (1) $p{Y}_{0}+q{Y}_{1}+{(L}_{k}-p{Y}_{0}-q{Y}_{1})(k-p-q)={L}_{\text{k}}$, (2) if $k-p-q>0$, then ${{Y}_{1}<L}_{k}-p{Y}_{0}-q{Y}_{1}<{Y}_{0}$.

We give intermediate quantities ${p}_{0}$ and ${q}_{0}$:

$${p}_{0}=\frac{{L}_{k}-k{Y}_{1}}{{Y}_{0}-{Y}_{1}}$$

$${q}_{0}=\frac{k{Y}_{0}-{L}_{k}}{{Y}_{0}-{Y}_{1}}$$

Calculation yields:

$${p}_{0}+{q}_{0}=k$$

$${p}_{0}{Y}_{0}+{q}_{0}{Y}_{1}={L}_{k}$$

If ${p}_{0}$ and ${q}_{0}$ are both integers, we can get $p={p}_{0}$, $q={q}_{0}$, ${k-p}_{0}-{q}_{0}=0$, and naturally we can prove $p{Y}_{0}+q{Y}_{1}+{(L}_{k}-p{Y}_{0}-q{Y}_{1})(k-p-q)={L}_{k}$, and all cut steel billets conform to the user’s target range.

If at least one of ${p}_{0}$ and ${q}_{0}$ is not an integer, according to ${p}_{0}+{q}_{0}=k$ and $k$ is an integer, we can deduce that ${p}_{0}$ and ${q}_{0}$ must both be non-integers. According to the definition of floor function, we can get $0<{p}_{0}+{q}_{0}-(p+q)<2$. And since ${p}_{0}+{q}_{0}=k$ and $k$ is an integer, we can get $k-p-q=1$ and ${p}_{0}+{q}_{0}-(p+q)=1$, thus:

$${L}_{k}-p{Y}_{0}-q{Y}_{1}={p}_{0}{Y}_{0}+{q}_{0}{Y}_{1}-p{Y}_{0}-q{Y}_{1}=({p}_{0}-p){Y}_{0}+({q}_{0}-q){Y}_{1}=({p}_{0}-p){Y}_{0}+(1-({p}_{0}-p)){Y}_{1}$$

Combined with ${0<p}_{0}-p<1$ At this point, we can get ${Y}_{1}<{L}_{k}-p{Y}_{0}-q{Y}_{1}<{Y}_{0}$.

Next, prove that it is not possible to cut $p+1$ steel billets according to ${Y}_{0}$.

According to the definition of floor function, $p+1>{p}_{0}$. We assume cutting $p+1$ steel billets according to ${Y}_{0}$, and cutting the remaining $k-(p+1)$ steel billets according to the shortest cuttable length (user’s target range lower limit) ${Y}_{1}$. Combined with ${p}_{0}{Y}_{0}+{q}_{0}{Y}_{1}={L}_{k}$, ${p}_{0}+{q}_{0}=k$, ${Y}_{0}>{Y}_{1}$, we can calculate:

$$(p+1){Y}_{0}+[k-(p+1)]{Y}_{1}-{L}_{k}=(p+1){Y}_{0}+[k-(p+1)]{Y}_{1}-({p}_{0}{Y}_{0}+{q}_{0}{Y}_{1})=(p+1-{p}_{0})({Y}_{0}-{Y}_{1})>0$$

That is:

$$(p+1){Y}_{0}+[k-(p+1)]{Y}_{1}>{L}_{k}$$

In other words, cutting $p+1$ steel billets according to ${Y}_{0}$ and cutting the remaining steel billets according to the shortest feasible length ${Y}_{1}$ will still exceed the total length ${L}_{\text{k}}$, i.e., cutting $p+1$ steel billets according to ${Y}_{0}$ is not feasible.

**Case 2:** $${{k}}{{{Y}}}_{0}{<{{L}}}_{{{k}}}\le {{k}}{{{Y}}}_{2}$$

$$p = \left\lfloor {\frac{{kY_{2} - L_{k} }}{{Y_{2} - Y_{0} }}} \right\rfloor$$

$$q = \left\lfloor {\frac{{L_{k} - kY_{0} }}{{Y_{2} - Y_{0} }}} \right\rfloor$$

Cut $p$ steel billets according to ${Y}_{0}$, cut $q$ steel billets according to ${Y}_{2}$, cut $k-p-q$ steel billets according to ${L}_{k}-p{Y}_{0}-q{Y}_{2}$, $D(L)=p$.

The proof for Case 2 is similar to Case 1.

**Case 3:** $${{k}}{{{Y}}}_{2}{<{{L}}}_{{{k}}}\le 12.6{{k}}$$

$$p = \left\lfloor {\frac{{12.6k - L_{k} }}{{12.6 - Y_{2} }}} \right\rfloor$$

$$q = \left\lfloor {\frac{{L_{k} - kY_{2} }}{{12.6 - Y_{2} }}} \right\rfloor$$

Cut $p$ steel billets according to ${Y}_{2}$, cut $q$ steel billets according to 12.6, cut $k-p-q$ steel billets according to ${L}_{k}-p{Y}_{2}-12.6q$, $D(L)=0$.

The proof for Case 3 is similar to Case 1.

#### Specific cutting scheme for the ${{{W}}}_{{{n}}}$ part

The optimal value of ${W}_{n}$ has already been found in the traversal process (obtained through the finally selected $k$ value and the finally selected sub-case, see 2.2.1 for details). If ${W}_{n}\ne 0$, we need to consider the cutting scheme for the ${W}_{n}$ part. Since we have obtained the optimal ${L}_{k}$ and ${W}_{n}$ through traversal, ${W}_{n}$ must be $<{Y}_{1}+4.8$ (otherwise, another usable steel billet could be cut out from the ${W}_{n}$ part, and it would not be the optimal ${L}_{k}$). We determine the cutting scheme and the value of $n$ according to the length of ${W}_{n}$: if ${0<W}_{n}\le 12.6$, cut ${W}_{n}$ into one steel billet, At this point $n=1$; if $12.6<{W}_{n}<{Y}_{1}+4.8$, cut ${W}_{n}$ into two steel billets of 4.8 and ${W}_{n}-4.8$, At this point $n=$ 2.

#### Optimization results for static billet tail cutting

Table 2 presents the optimal cutting schemes for several billet tail lengths given in the competition problem, with the user target length set as $Y_{0} = 9.5$, the lower limit as $Y_{1} = 9.0$, and the upper limit as $Y_{2} = 10.0$. However, our research is not limited to solving only the example data provided in the competition. The developed algorithm can compute optimal cutting schemes for any billet tail length and any user target requirements within the permissible process range. Users can input their specific parameters into the calculation software developed by the authors to obtain corresponding results. The software sharing link, along with the online optimization software for real-time adjustments, is provided in Section "Interactive demonstration for arbitrary input data".

The optimal cutting schemes for different billet tail lengths under these specific user requirements are summarized in Table 2.

**Table 2:** Cutting schemes for billet tails of different lengths.

Billet tails length	Cutting scheme	Cutting loss
109.0	9.0, 9.0, 9.0, 9.0, 9.0, 9.0, 9.0, 9.0, 9.0, 9.0, 9.5, 9.5	0
93.4	9.4, 9.0, 9.0, 9.0, 9.5, 9.5, 9.5, 9.5, 9.5, 9.5	0
80.9	10.0 + 0.9, 10.0, 10.0, 10.0, 10.0, 10.0, 10.0, 10.0	0.9
72.0	9.0, 9.0, 9.0, 9.0, 9.0, 9.0, 9.0, 9.0	0
62.7	10.0 + 0.1, 10.0 + 2.6, 10.0, 10.0, 10.0, 10.0	2.7
52.5	10.0 + 2.5, 10.0, 10.0, 10.0, 10.0	2.5
44.9	4.9, 10.0, 10.0, 10.0, 10.0	4.9
42.7	10.0 + 0.1, 10.0 + 2.6, 10.0, 10.0	2.7
31.6	10.0 + 1.6, 10.0, 10.0	1.6
22.7	10.0 + 0.1, 10.0 + 2.6	2.7
14.5	4.8, 9.7	4.8
13.7	8.9, 4.8	13.7

**Fig. 4:**
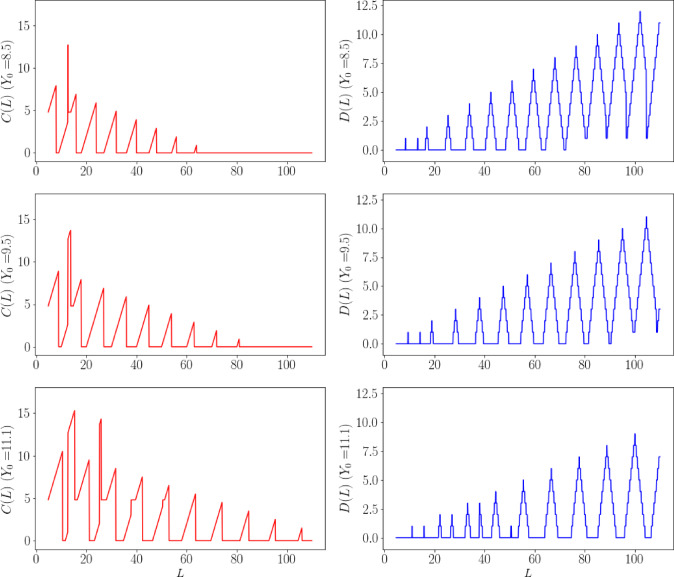
$C(L)$ and $D(L)$ function graph.

We have drawn the minimum cutting loss function $C(L)$ and the function $D(L)$ for the maximum number of steel billets equal to the user’s target value under the three user requirements in the problem, as shown in Fig. 4.

### Cutting model for "sub-intervals to be planned" containing abnormal scrap sections (sub-model)

**Fig. 5:**
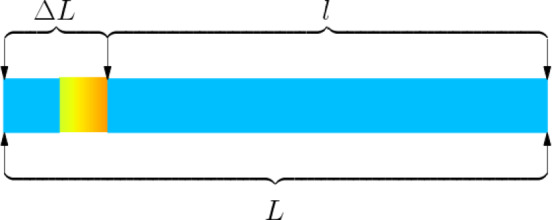
Schematic diagram of sub-interval to be planned.

We now begin to address Problems 2 and 3 (dynamic optimization problems with abnormal segments). First, we establish a model to solve the cutting scheme for a "sub-interval to be planned" containing an abnormal segment, as shown in Fig. 5.

Our model needs to consider two properties: (1) There exists a feasible solution for the cutting scheme of this "sub-interval to be planned $L$"; (2) The $\Delta L$ part can only be entirely scrapped. Therefore, there are certain requirements for the length range of $L$ and $\Delta L$, specifically as follows:

$$\left\{\begin{array}{c}L\ge 4.8\\ 0.8\le \Delta L<{Y}_{1}+0.8\\ \Delta L\le L\end{array}\right.$$

This is the applicable range of our model. Within this range, we solve for three cases.

**Case 1**

**Case 1 Identification:**

First, we determine whether it belongs to Case 1 through calculation. The calculation steps are as follows: As shown in Fig. 5, for a "sub-interval to be planned" containing an abnormal segment, we assume cutting $L$ according to the billet tail model (including online and offline cutting). If the cutting result contains $N$ scrapped steel billets, and $N\ne 0$, we record the length of each scrapped steel billet as ${a}_{i}(i=\text{1,2},3\cdots N)$. If

$$\text{max}({a}_{i})\ge\Delta L$$

then it is determined to belong to Case 1.

**Case 1 Scheme:**

First, calculate the cutting scheme of *L* according to the billet tail model. Then, adjust the order of online cutting according to the cutting loss of each steel billet. Move the steel billet with the maximum cutting loss (if it is a single scrapped steel billet, the cutting loss is the length of this steel billet; if it is a steel billet planned for offline cutting, the cutting loss is the length of this steel billet planned to be scrapped after offline cutting) to the end. After adjusting the order of online cutting in the billet tail model, we can obtain the cutting scheme for Case 1.

${C}_{1}(L,\Delta L)$ is the minimum cutting loss of the sub-interval when $L$ and $\Delta L$ conform to Case 1. We calculate ${C}_{1}(L,\Delta L)$, and obviously, ${C}_{1}(L,\Delta L)=C(L)$.

**Proof of Case 1:**

According to the billet tail cutting model, changing the order of each steel billet obtained during online cutting does not affect the optimization objectives at all levels. For example, a 21-m billet tail can be cut online into 10 m first and then 11 m, or it can be cut online into 11 m first and then 10 m. The optimization indicators at all levels for both schemes are the same.

In our Case 1 scheme, we only adjust the order of the online cutting stage in the billet tail model, so the abnormal segment naturally falls within the scrapped steel billet. Therefore, our Case 1 cutting scheme is equivalent to the billet tail model cutting scheme and is the optimal scheme.

**Case 2**

**Case 2 Identification:**

First, we determine whether it belongs to Case 2 through calculation. The calculation steps are as follows: As shown in Fig. 5, $l=L-\Delta L$. We assume cutting the l part online according to the billet tail cutting scheme (excluding offline cutting). Suppose it is cut into N steel billets, and the length of each steel billet is recorded as ${b}_{i}(i=\text{1,2},3\cdots N)$. If ${b}_{i}\ge {y}_{1}$, we record $12.6-{b}_{i}$ as ${h}_{i}$.

If the following two conditions are met, it is determined to belong to Case 2:

- $$\text{max}({h}_{i})\ge\Delta L$$
- The combination of $L$ and $\Delta L$ does not conform to Case 1 in this section.

If the above conditions are met, it is Case 2. We further divide Case 2 into two sub-cases.

**Sub-case 2.1 Identification:**

When cutting l according to the billet tail model, the final selected k value and cutting scheme do not correspond to Case 2.1 in Section "[Sec Sec17]".

**Sub-case 2.2 Identification:**

When cutting l according to the billet tail model, the final selected k value and cutting scheme correspond to Case 2.1 in Section "[Sec Sec17]".

**Sub-case 2.1 Scheme:**

1. First, take out the $\Delta L$ part separately.
2. Cut the remaining $l$ part online according to the billet tail cutting scheme, then adjust the cutting order, moving the steel billet corresponding to $\text{max}({h}_{i})$ to the end, and attach the $\Delta L$ part to this steel billet.
3. Regarding the special offline cutting of the steel billet with the attached $\Delta L$ part, let the length of this steel billet before attaching $\Delta L$ be ${b}_{L}$. If ${b}_{L}<{Y}_{2}$, cut it offline into two parts: ${b}_{L}$ and $\Delta L$. If ${b}_{L}\ge {Y}_{2}$, cut it offline into two parts: ${Y}_{2}$ and ${b}_{L}+\Delta L-{Y}_{2}$.
4. Let the total cutting loss caused by offline cutting At this point be ${C}_{V}$, and the total cutting loss of single scrapping be ${C}_{U}$ (if there is no single scrapping, then ${C}_{U}=0$). If $4.8\le {C}_{V}+{C}_{U}$, combine all parts of cutting loss into 1 steel billet; otherwise, leave it unchanged.

${C}_{21}(L,\Delta L)$ is the minimum cutting loss of the sub-interval when $L$ and $\Delta L$ conform to Sub-case 2.1. We calculate ${C}_{21}(L,\Delta L)$, and obviously, ${C}_{21}(L,\Delta L)=C(L-\Delta L)+\Delta L$.

**Sub-case 2.2 Scheme:**

Suppose the l part can produce k usable steel billets when cut according to the billet tail model. After program verification for all user target requirements ${Y}_{0}\in [\text{8.5,11.1}]$ and all combinations of $L$ and $\Delta L$, Case 2.2 only exists when $k=1$, that is: ${Y}_{1}+4.8\le l<{Y}_{2}+4.8$. Because it conforms to Case 2, obviously $\Delta L\le 12.6-(l-4.8)$. Our scheme is as follows: If $L-{Y}_{2}<4.8$, cut L into two steel billets of $L-4.8$ and $4.8$. If $L-{Y}_{2}\ge 4.8$, cut $L$ into two steel billets of ${Y}_{2}$ and $L-{Y}_{2}$.

${\text{C}}_{22}(L,\Delta L)$ is the minimum cutting loss of the sub-interval when $L$ and $\Delta L$ conform to Sub-case 2.2. We calculate ${\text{C}}_{22}(L,\Delta L)$, and obviously, ${\text{C}}_{22}(L,\Delta L)=\text{max}({L-Y}_{2},4.8)$.

**Proof of Case 2:**

We have provided proofs for Sub-case 2.1 satisfying the three-level optimization objectives of "minimum cutting loss", "maximum number of steel billets equal to the user’s target value", and "minimum number of offline cuttings". The proof is lengthy and can be found in Appendix.

For Sub-case 2.2, our scheme is obviously a feasible solution under all the identification conditions of this case, and it is the optimal solution.

**Case 3**

**Case 3 Identification:**

$L\le 12.6$, and it does not belong to Case 1 or Case 2, then it is determined to be Case 3.

**Case 3 Scheme:**

Cut $L$ into one (scrapped) steel billet.

${C}_{3}(L,\Delta L)$ is the minimum cutting loss of the sub-interval when $L$ and $\Delta L$ conform to Case 3. We calculate ${C}_{3}(L,\Delta L)$, and obviously ${C}_{3}(L,\Delta L)=L$.

**Proof of Case 3:**

Under the premise of $L\le 12.6$: If $L-\Delta L\ge {Y}_{1}$, it obviously belongs to Case 2. If $L-\Delta L<{Y}_{1}$, according to the definition range of $\Delta L$ in this model, the $\Delta L$ part can only be scrapped, and the $L-\Delta L$ part, due to its length being less than ${Y}_{1}$, can also only be scrapped. Therefore, the entire $L$ can only be entirely scrapped.

**Fig. 6:**
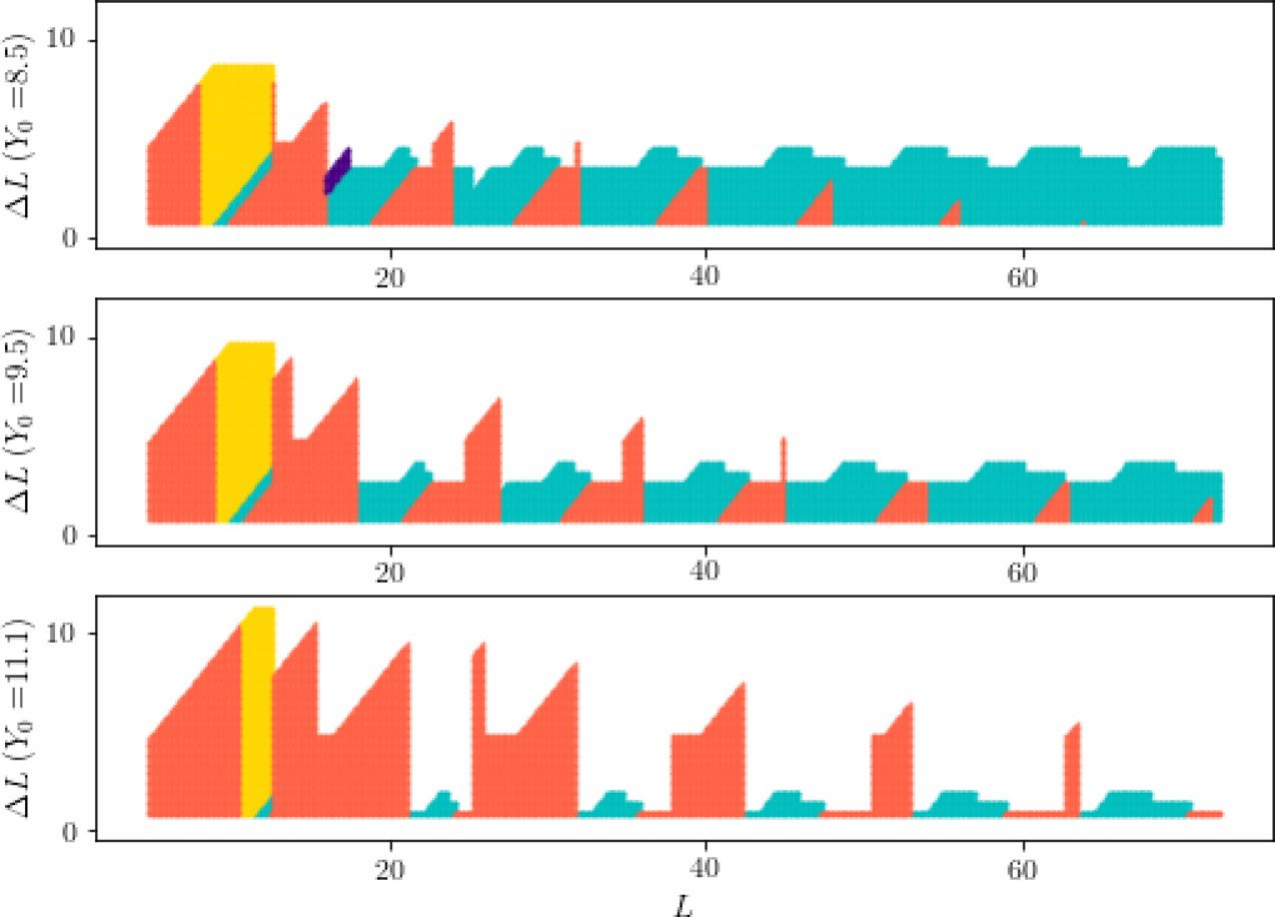
Coverage range diagram of three cases.

Next, we begin to measure the range of optimal solutions that our sub-model can achieve for all combinations of $L$ and $\Delta L$. If it conforms to Case 1 or Case 2 or Case 3, our model can obtain the optimal solution. For the three sets of user requirements in Problems 2 and 3 of the question, we have conducted computer verification and drawn graphics (Fig. 6). The light red part indicates conformity to Case 1, the light blue part indicates conformity to Sub-case 2.1, the purple part indicates conformity to Sub-case 2.2, and the light yellow part indicates conformity to Case 3.

Based on the above graphical analysis and program verification (to ensure the reliability of the conclusions, we have actually used the program to traverse and verify all user target values ${Y}_{0}\in \left[\text{8.5,11.1}\right]$ and all sub-interval lengths L where $4.8\le L\le 72.6$, and have done statistics), we obtained the following conclusions.

According to our sub-model calculation, for any combination of L and Δ*L* within the range of the sub-model:

1. When $L\le 12.6$, it must obtain the optimal solution.
2. When $\Delta L=0.8$, it must obtain the optimal solution.
3. When $\Delta L\le 12.6-{Y}_{2}$, for all possible combinations of $L$ and ${Y}_{0}$, 99.7% of cases can obtain the optimal solution.
4. When $\Delta L\le 12.6-{Y}_{1}$, for all possible combinations of $L$ and ${Y}_{0}$, 82.6% of cases can obtain the optimal solution.

On this basis, we establish the ${C}_{X}$ function, defined as follows:

If the combination of *L* and Δ*L* conforms to Case 1, then

$${C}_{X}(L,\Delta L)={C}_{1}(L,\Delta L)$$

If the combination of *L* and Δ*L* conforms to Case 2.1, then

$${C}_{X}(L,\Delta L)={C}_{21}(L,\Delta L)$$

If the combination of *L* and Δ*L* conforms to Case 2.2, then

$${C}_{X}(L,\Delta L)={C}_{22}(L,\Delta L)$$

If the combination of *L* and Δ*L* conforms to Case 3, then

$${C}_{X}(L,\Delta L)={C}_{3}(L,\Delta L)$$

For other cases, we stipulate

$${C}_{X}(L,\Delta L)=\infty$$

The purpose of establishing the ${C}_{X}$ function is to call this function in the main model in Section "Online Cutting Model (Main Model)".

### Online cutting model (main model)

#### Minimum cutting loss model

We now enter our main model. Our model approach is to establish a planning model, taking the boundary position of the "interval to be optimized" as the free variable of the planning model, and using the model in 2.1 as a sub-model (sub-expression) within the planning model, to solve the entire planning model.

Considering the online cutting process, when a new abnormal scrap section appears in the crystallizer, triggering an “alarm”, we begin to calculate a new round of cutting plans, specifically divided into the following situations.

**Situation 1**

There is 1 abnormal scrap section between the cutting machine and the crystallizer (including inside the crystallizer At this point, same below).

**Fig. 7:**
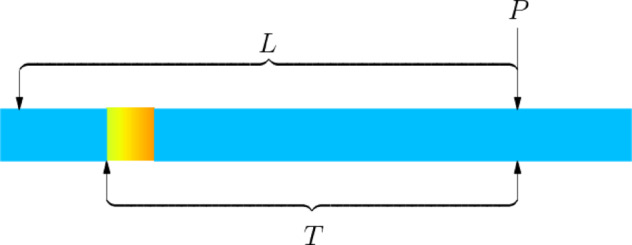
Schematic diagram of Situation 1.

As shown in Fig. 7, we will do single-interval planning for Situation 1, divided into 3 sub-situations according to different starting positions P of the planning.

**Sub-situation 1.1:**

The number of abnormal scrap sections between the crystallizer and the cutting machine $M=1$, and the cutting machine is currently cutting, then point $P$ is the current position of the cutting machine.

**Sub-situation 1.2:**

The number of abnormal scrap sections between the crystallizer and the cutting machine $M=1$, and the cutting machine is currently waiting to cut or returning to the starting point, then point $P$ is the position of the last cut of the cutting machine.

**Sub-situation 1.3:**

At this moment, it is in the initial stage of continuous casting, the starting point of the continuous casting billet has not reached the position of the cutting machine, the number of abnormal scrap sections between the crystallizer and the starting point of continuous casting $M=1$, then point P is the starting position of the entire continuous casting cutting (At this point there is no billet to the right of point $P$).

For sub-situation 1, we establish a planning model and calculate using the ${C}_{X}$ function in 2.1.

$$\text{s}.\text{t}. \left\{\begin{array}{c}L\in \left[\text{max}\left(4.8,T\right),T+{Y}_{1}\right)\\ \Delta L=L-T+0.8\end{array}\right.$$

$$min{C}_{Z}(L,\Delta L)=\text{min}[{C}_{X}(L,\Delta L)]$$

Since $\Delta L$ can be calculated from $L$ and the constant $T$, in essence, the above planning model has only one free variable $L$.

The computer traverses all $L$ within the feasible domain to obtain $min{C}_{Z}(L,\Delta L)$ and its corresponding $L$.

Additionally, for sub-situations 1.1 and 1.2, if based on the distance of 60 m between the crystallizer and the starting point of the cutting machine, and the user requirements in the problem, in fact, $L=T$, i.e., $\Delta L=0.8$ is the optimal sub-interval division method.

**Situation 2**

There are at least 2 abnormal scrap sections between the cutting machine and the crystallizer

**Fig. 8:**
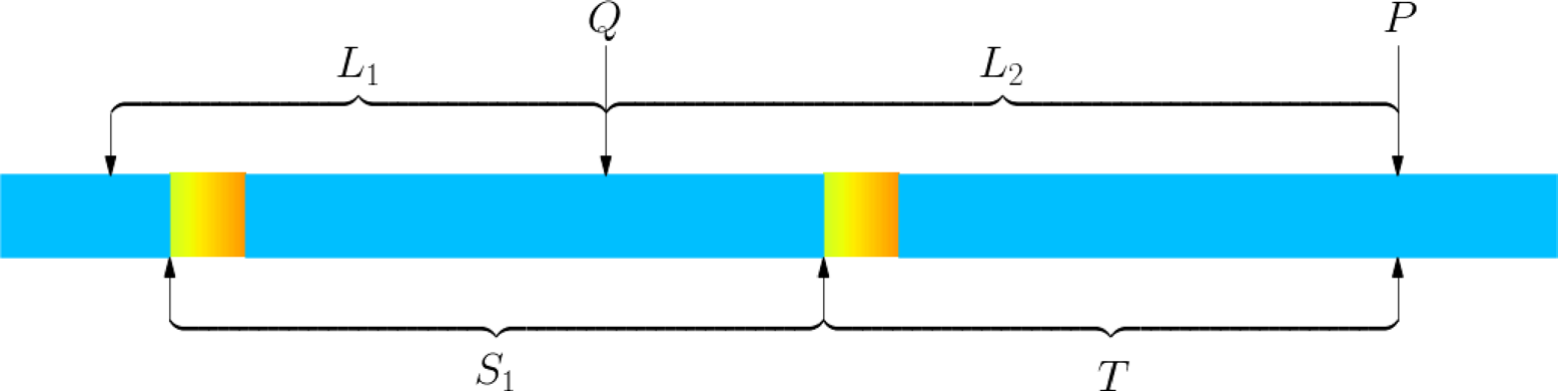
Schematic diagram of Situation 2.

As shown in Fig. 8, we will do double-interval planning for Situation 2. Point $P$ is the starting position of the planning, and the specific position of point $P$ is divided into 4 sub-situations.

**Sub-situation 2.1:**

The number of abnormal scrap sections between the crystallizer and the cutting machine $M=2$, and the cutting machine is currently cutting, then point $P$ is the current position of the cutting machine.

**Sub-situation 2.2:**

The number of abnormal scrap sections between the crystallizer and the cutting machine $M=2$, and the cutting machine is currently waiting to cut or returning to the starting point, then point $P$ is the position of the last cut of the cutting machine.

**Sub-situation 2.3:**

The number of abnormal scrap sections between the crystallizer and the cutting machine $M>2$, then point $P$ is the "$Q$ point" position in the previous round of planning.

**Sub-situation 2.4:**

At this moment, it is in the initial stage of continuous casting, the starting point of the continuous casting billet has not reached the position of the cutting machine, the number of abnormal scrap sections between the crystallizer and the starting point of continuous casting $M=2$, then point $P$ is the starting position of the entire continuous casting cutting (At this point there is no billet to the right of point $P$).

Special note on the planning starting position for sub-situations 1.2 and 2.2: If after the new plan is generated, it is found that the position of the first cut in the new plan has already passed the starting point of the cutting machine’s work, making it impossible to make the first cut, then re-plan after making 1 more cut according to the previous round of planning.

For Situation 2, we establish a planning model and calculate using the ${C}_{X}$ function in 2.1, where ${C}_{Z}$ is the sum of the minimum cutting losses of the two sub-intervals ${L}_{1}$ and ${L}_{1}$ based on given ${L}_{1},{L}_{2},\Delta {L}_{1},\Delta {L}_{2}$.

$$\text{s}.\text{t}. \left\{\begin{array}{c}{L}_{1}\in \left[\text{max}\left(4.8,T+{S}_{1}-{L}_{2}\right),T+{S}_{1}-{L}_{2}+{Y}_{1}\right)\\ {L}_{2}\ge \text{max}\left(4.8,T\right)\\ {L}_{2}\le T+{S}_{1}-0.8\\ {L}_{2}<T+{Y}_{1}\\ \Delta {L}_{1}={L}_{1}+{L}_{2}-{S}_{1}-T+0.8\\ \Delta {L}_{2}={L}_{2}-T\\ {C}_{Z}\left({L}_{1},{L}_{2},\Delta {L}_{1},\Delta {L}_{2}\right)={C}_{X}\left({L}_{1},\Delta {L}_{1}\right)+{C}_{X}\left({L}_{2},\Delta {L}_{2}\right)\end{array}\right.$$

$$min{C}_{Z}({L}_{1},{L}_{2},\Delta {L}_{1},\Delta {L}_{2})=\text{min}[{C}_{Z}({L}_{1},{L}_{2},\Delta {L}_{1},\Delta {L}_{2})]$$

Since $\Delta {L}_{1},\Delta {L}_{2}$ can be calculated from ${L}_{1},{L}_{2}$ and constants $T,{S}_{1}$, in essence, the above planning model has only two free variables, namely ${L}_{1},{L}_{2}$.

The computer traverses all ${L}_{1},{L}_{2}$ within the feasible domain to obtain $min{C}_{Z}({L}_{1},{L}_{2},\Delta {L}_{1},\Delta {L}_{2})$ and its corresponding ${L}_{1},{L}_{2}$.

For some special cases that cause the feasible domain of $L_{1}$ or $L_{2}$ to be $\emptyset$, the handling methods are as follows.

**Special Case 1:** Invalid abnormal segment.

**Fig. 9:**
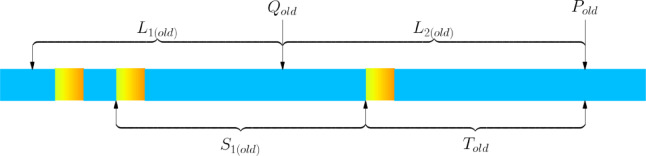
Schematic diagram of Special Case 1.

As shown in Fig. 9, if the newly appeared abnormal segment is within the ${L}_{1}$ range of the previous round of planning, it is an "invalid abnormal segment" and does not trigger a new round of planning.

**Special Case 2:** $$\left\{\begin{array}{c}{{{S}}}_{1}\ge 0.8\\ {{{S}}}_{1}+T<5.6\end{array}\right.$$

**Fig. 10:**
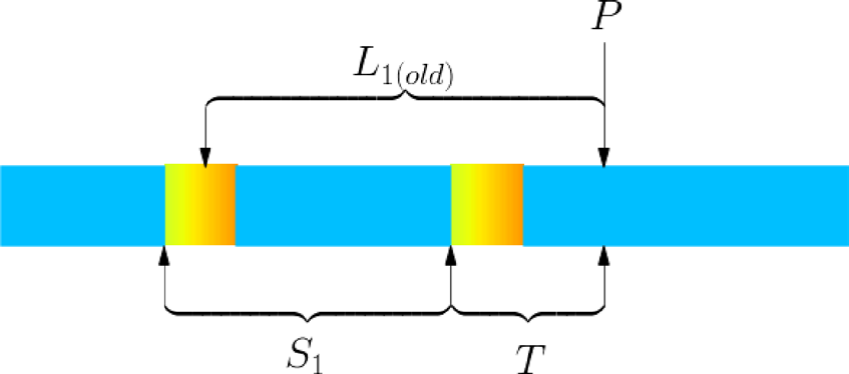
Schematic diagram of Special Case 2.

The schematic representation of this case is shown in Fig. 10.

**Special Sub-case 2.1** **:** ${{{S}}}_{1}+{{T}}\le 4.8$

Since ${L}_{1(old)}\ge 4.8$, this sub-case is essentially a subset of Special Case 1 and can be handled as Special Case 1.

**Special Sub-case 2.2:** $4.8<{{{S}}}_{1}+{{T}}<5.6$

For this case, we calculate in this round of the main model with ${S}_{1}=5.6-T$. At this point, it will inevitably result in ${L}_{1},{L}_{2}$ both being 4.8 corresponding to $min{C}_{Z}$. Later, in the post-optimization stage of the main model results in Section "Further optimization of the cutting plan", ${L}_{1},{L}_{2}$ will be combined into a single steel billet with a length of 9.6 and then shortened to a steel billet with a length of ${S}_{1}+T$, which is already the optimal result.

**Special Case 3:** Overlapping abnormal segments $({S}_{1}<0.8)$

**Fig. 11:**
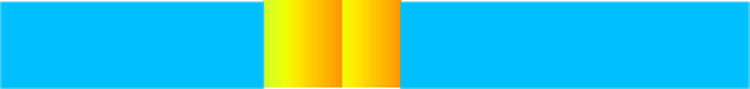
Schematic diagram of Special Sub-case 3.1

**Special Sub-case 3.1:** Two abnormal segments partially overlap,The schematic representation of this case is shown in Fig. 11.

**Special Sub-case 3.1.1:** ${{{S}}}_{1}+{{T}}\le 4.8$

This sub-case is a subset of Special Case 1 and can be handled as Special Case 1.

**Special Sub-case 3.1.2:** $4.8<{{{S}}}_{1}+{{T}}<5.6$

We calculate in this round of the main model with ${S}_{1}=0.8,T=4.8$. The calculation result is the same as Special Sub-case 2.2, which is already the optimal result.

**Special Sub-case 3.1.3:** ${{{S}}}_{1}+{{T}}\ge 5.6$

We calculate in this round of the main model with ${S}_{1}=0.8$, which will inevitably result in ${L}_{1}=4.8,{L}_{2}=T$, which is already the optimal result.

**Fig. 12:**
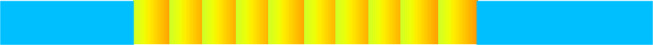
Schematic diagram of Special Sub-case 3.2

**Special Sub-case 3.2:** Multiple consecutive abnormal segments partially overlap, The schematic representation of this case is shown in Fig. 12.

In this case, if the first and second abnormal segments are both valid abnormal segments, then the third abnormal segment must be an invalid abnormal segment. In other words, there can be at most 2 consecutive overlapping valid abnormal segments, and the model will still automatically calculate according to "Special Sub-case 3.1" to obtain the correct result.

Proof that the main model must have a solution ($min{C}_{Z}$ will not solve to $\infty$): If the lower limit of the length in the feasible domain of ${L}_{1}$ or ${L}_{2}$ is greater than 4.8, then there exists at least one feasible solution where $\Delta {L}_{1}$ or ${\Delta L}_{2}$ equals 0.8. If the lower limit of the length in the feasible domain of ${L}_{1}$ or ${L}_{2}$ equals 4.8, then there exists at least one feasible solution where ${L}_{1}$ or ${L}_{2}$ equals 4.8.

#### Second and third level objective optimization model

Assuming that under the premise of achieving minimum cutting loss, the maximum number of steel billets equal to the user’s target value is $max{D}_{Z}({L}_{1},{L}_{2},\Delta {L}_{1},\Delta {L}_{2})$, and under the premise of satisfying minimum cutting loss and maximum number of steel billets equal to the user’s target value, the minimum number of offline cuttings is max $max{E}_{Z}({L}_{1},{L}_{2},\Delta {L}_{1},\Delta {L}_{2})$. The ${L}_{1},{L}_{2}$ corresponding to $min{C}_{Z}$ may be unique or not unique. If they are not unique, we set any two groups of ${L}_{1},{L}_{2}$ that achieve $min{C}_{Z}$ as specific solutions ${L}_{11},{L}_{12}$ and ${L}_{21},{L}_{22}$. If the following situations exist, we need to consider ${L}_{1},{L}_{2}$ corresponding to the second and third level optimization objectives.

**Situation 1:**

$$\left\{\begin{array}{c}min{C}_{Z}({L}_{1},{L}_{2},\Delta {L}_{1},\Delta {L}_{2})={C}_{Z}({L}_{11},{L}_{12},\Delta {L}_{11},\Delta {L}_{12})={C}_{Z}({L}_{11},{L}_{12},\Delta {L}_{11},\Delta {L}_{12})\\ {D}_{Z}({L}_{11},{L}_{12},\Delta {L}_{11},\Delta {L}_{12})\ne {D}_{Z}({L}_{11},{L}_{12},\Delta {L}_{11},\Delta {L}_{12})\end{array}\right.$$

If Situation 1 exists, we need to compare the ${D}_{Z}$ of all ${L}_{1},{L}_{2}$ that achieve $\text{min}{C}_{Z}$. For this, our method is to collect all ${L}_{1},{L}_{2}$ that achieve the minimum $min{C}_{Z}$, and traverse them to calculate max $max{D}_{Z}$, thus obtaining the ${L}_{1},{L}_{2}$ corresponding to $max{D}_{Z}$.

**Situation 2:**

$$\left\{\begin{array}{c}min{C}_{Z}({L}_{1},{L}_{2},\Delta {L}_{1},\Delta {L}_{2})={C}_{Z}({L}_{11},{L}_{12},\Delta {L}_{11},\Delta {L}_{12})={C}_{Z}({L}_{11},{L}_{12},\Delta {L}_{11},\Delta {L}_{12})\\ max{E}_{Z}({L}_{1},{L}_{2},\Delta {L}_{1},\Delta {L}_{2})={D}_{Z}({L}_{11},{L}_{12},\Delta {L}_{11},\Delta {L}_{12})={D}_{Z}({L}_{11},{L}_{12},\Delta {L}_{11},\Delta {L}_{12})\\ {E}_{Z}({L}_{11},{L}_{12},\Delta {L}_{11},\Delta {L}_{12})\ne {E}_{Z}({L}_{11},{L}_{12},\Delta {L}_{11},\Delta {L}_{12})\end{array}\right.$$

If Situation 2 exists, we need to compare the $max{E}_{Z}$ of all ${L}_{1},{L}_{2}$ with the same first two level indicators. For this, our method is to collect all L₁, L₂ that achieve the minimum $min{C}_{Z}$ and $max{D}_{Z}$, and traverse them to calculate $min{E}_{Z}$, thus obtaining the ${L}_{1},{L}_{2}$ corresponding to $min{E}_{Z}$.

After finding the optimal ${L}_{1},{L}_{2}$, substitute ${L}_{1},{L}_{2}$ into the sub-model in Section "Competition problem definition and optimization problem definition" to obtain the specific cutting scheme.

**Fig. 13:**
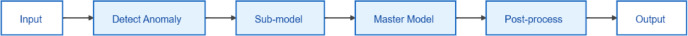
Online Optimization Flowchart.

Figure 13 illustrates the complete workflow of the online optimization algorithm, which is divided into six steps:

1. *Input*: Read cutter position $T$, anomaly coordinates {${S}_{i}$}, and user parameters $(Y_{0} ,Y_{1} ,Y_{2} )$.
2. *Detect anomaly*: Count anomaly segments $M$: $M=1$→single interval;$M\ge 2$→double interval.
3. *Sub-model*: For each ($L,\Delta L$), invoke Section "Cutting model for sub-intervals to be planned containing abnormal scrap sections (sub-model)" $C_{{\text{x}}} \left( {L,{\Delta }L} \right)$ to compute minimal cutting loss.
4. *Master model*: Enumerate free variable(s) $L$ (and $L_{2}$), solve $C_{Z} = min C_{{\text{x}}}$; if ties, filter by Level 2 $(\max\,{D}_{Z})$ and Level 3 (min ${E}_{Z}$) per Section "Second and third level objective optimization model".
5. *Post-process*: Apply Section "Further optimization of the cutting plan"’s merging/refinement: cross-interval merge, scrap merge, tail micro-adjustment ($\ge 4.8 m$).
6. *Output*: Produce final online‐optimal cut lengths {$L_{1} ,L_{2} \ldots$}, dispatch to cutter, and loop for next optimization.

#### Further optimization of the cutting plan

After calculation by the main model, we obtain the optimal cutting scheme of the main model. However, considering that sometimes the steel billets in ${L}_{1}$ can be combined with the steel billets in ${L}_{2}$ into 1 (cross-sub-interval combination) to reduce the number of cuts, we make further optimizations for the following 3 situations.

**Situation 1:**

Assuming that the overall cutting scheme obtained by the main model has N steel billets, if two adjacent steel billets are both scrapped steel billets (length does not meet usable requirements or contains abnormal segments), and the sum of their lengths $\le 12.6$, combine them.

**Situation 2:**

If the $i$-th steel billet and the ($i+2$)-th steel billet meet the combination conditions, and both the ($i+1$)-th and the ($i+2$)-th do not contain abnormal segments, exchange the order of the ($i+1$)-th and the ($i+2$)-th, then combine the $i$-th and the original ($i+2$)-th.

**Situation 3:**

After combining according to Situations 1 and 2, if the length of the last steel billet (which must contain the last (newest) abnormal segment) is $\ge 4.8$ and the position of the abnormal segment is not at the end of this steel billet, let the total length of the abnormal segment and the part before the abnormal segment in this steel billet be ${r}_{l}$, we shorten the length of this steel billet to $\text{max}(4.8,{r}_{l})$.

### Visualization and analysis of results

#### Cutting optimization results based on problem data

Below is the cutting plan based on the data for Questions 2 and 3. The cutting situation of the cutting machine at the initial moment is not given in the question, it can be within a reasonable range. In this paper, we set the first planning starting point at 0 (60 m away from the first abnormal scrap section).

Problems 2 and 3 are dynamic optimization problems. The types of the two problems are the same, with the only difference being in the user’s target requirements. After multiple rounds of online optimization, the final cutting situation is as shown in the figure below.

**Fig. 14:**
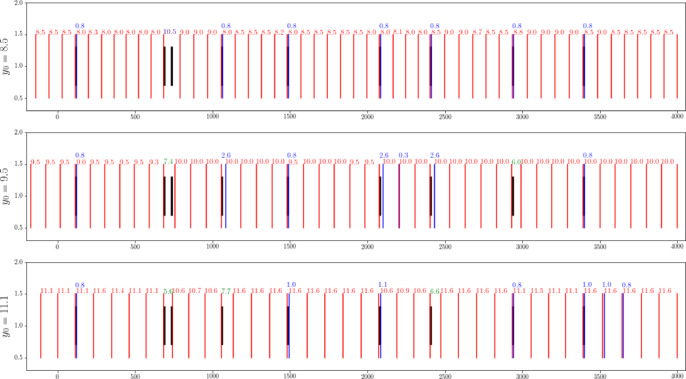
Overview of cutting schemes for Problems 2 and 3.

Figure 14 presents the final results after multiple rounds of online optimization in a static manner. If you need to understand the online optimization process as abnormal segments appear successively, you can input the data into our dynamic demonstration software for a dynamic demonstration, as detailed in Section "Interactive demonstration for arbitrary input data".

#### Interactive demonstration for arbitrary input data

We have developed three software applications in EXE format for computing optimal cutting schemes and dynamically demonstrating the online optimization process. These applications support user-defined data input and perform fully automated computations. They have been shared via Figshare and can be accessed using the following DOI: 10.6084/m9.figshare.27854517.

**Fig. 15:**
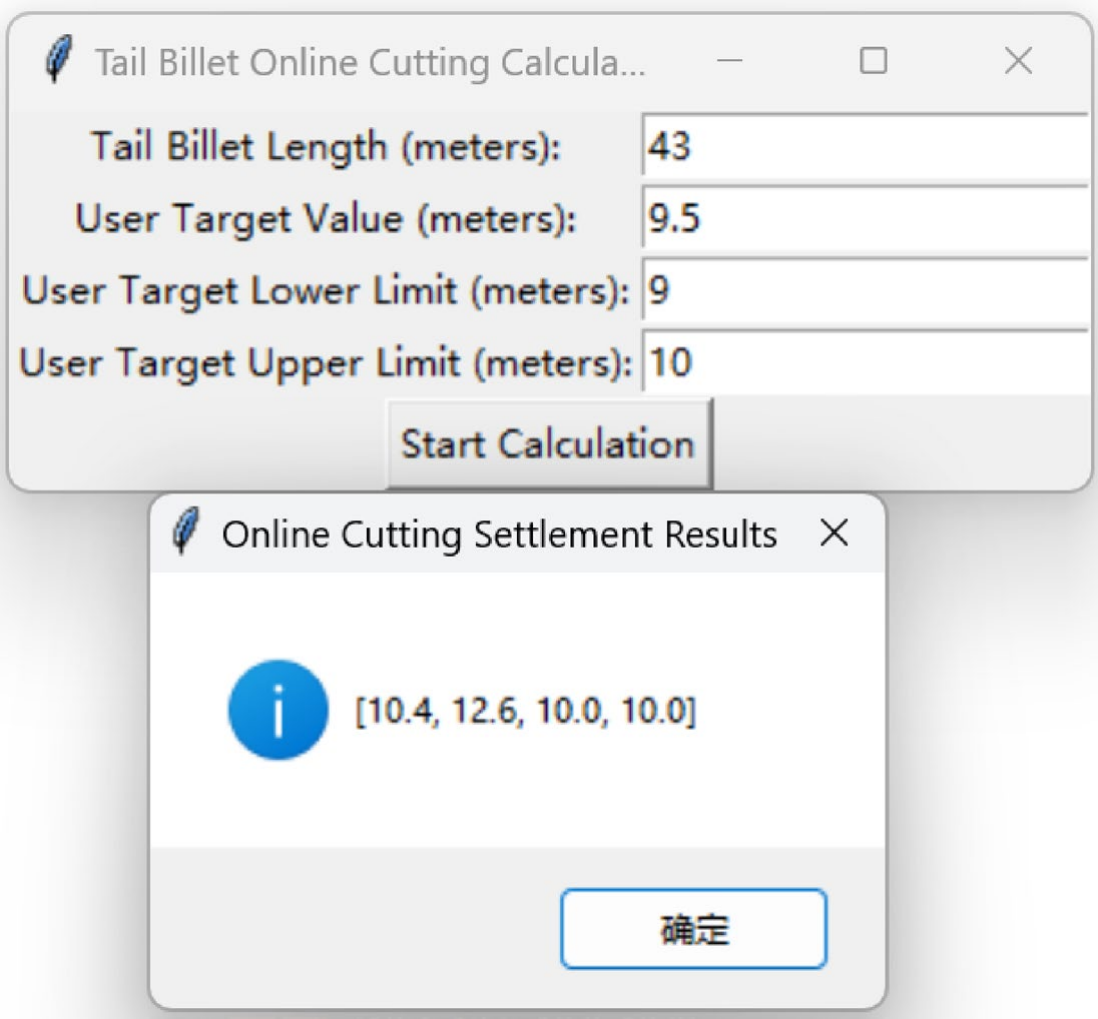
Static optimization problem (billet tail cutting) calculation software.

The first software is for the static optimization problem. After inputting the billet tail length and clicking calculate, the software gives the cutting scheme, as shown in Fig. 15.

**Fig. 16:**
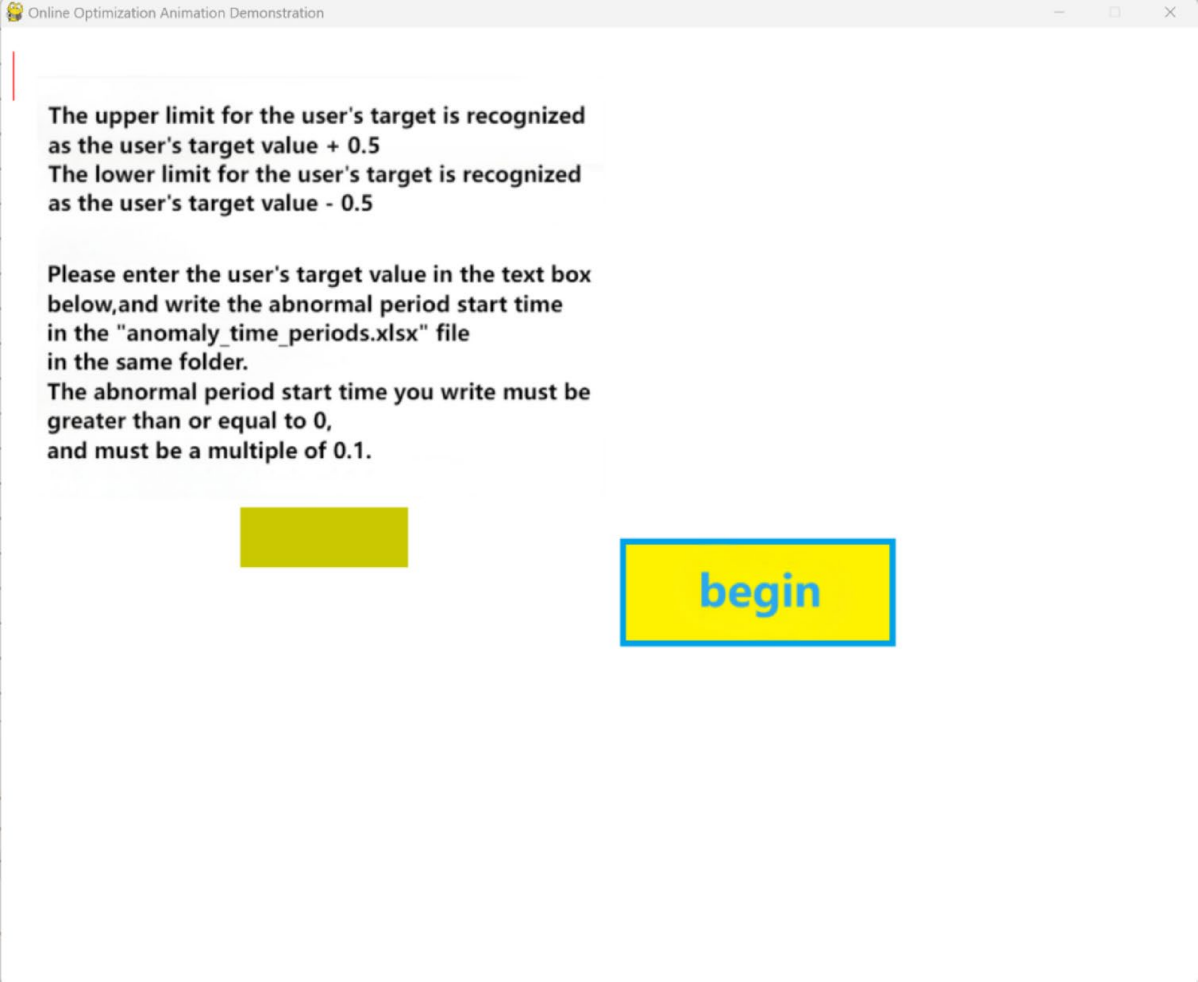
Preset data software input parameter interface.

**Fig. 17:**
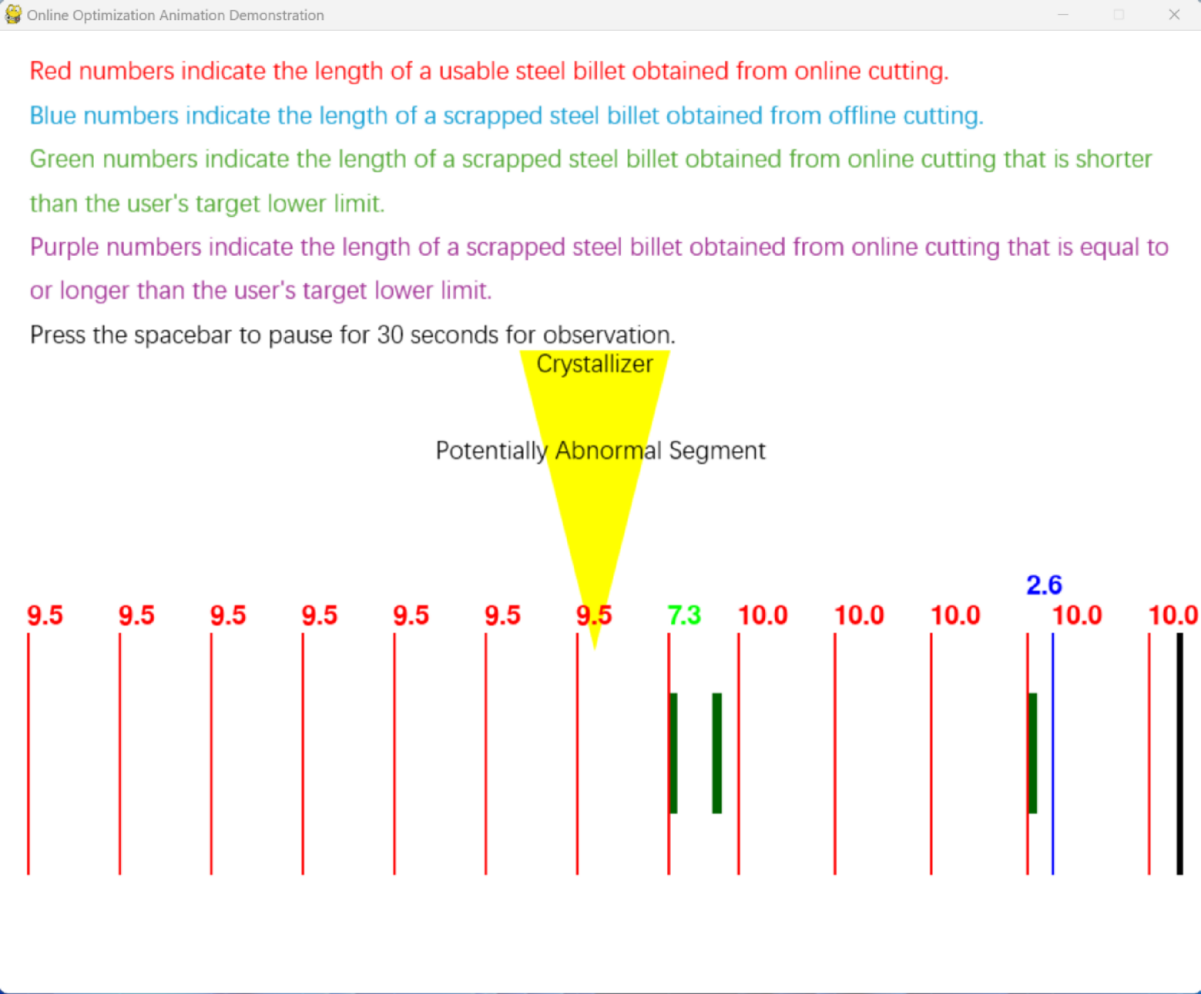
Preset data software animation demonstration interface.

There are two dynamic demonstration software applications. The first one inputs the preset occurrence times of each abnormal segment into an Excel spreadsheet (anomaly_time_periods) and inputs the user requirements into the software interface. After clicking start, the software will demonstrate the entire online cutting and real-time optimization process in the form of animation, as shown in Figs. 16 and 17.

**Fig. 18:**
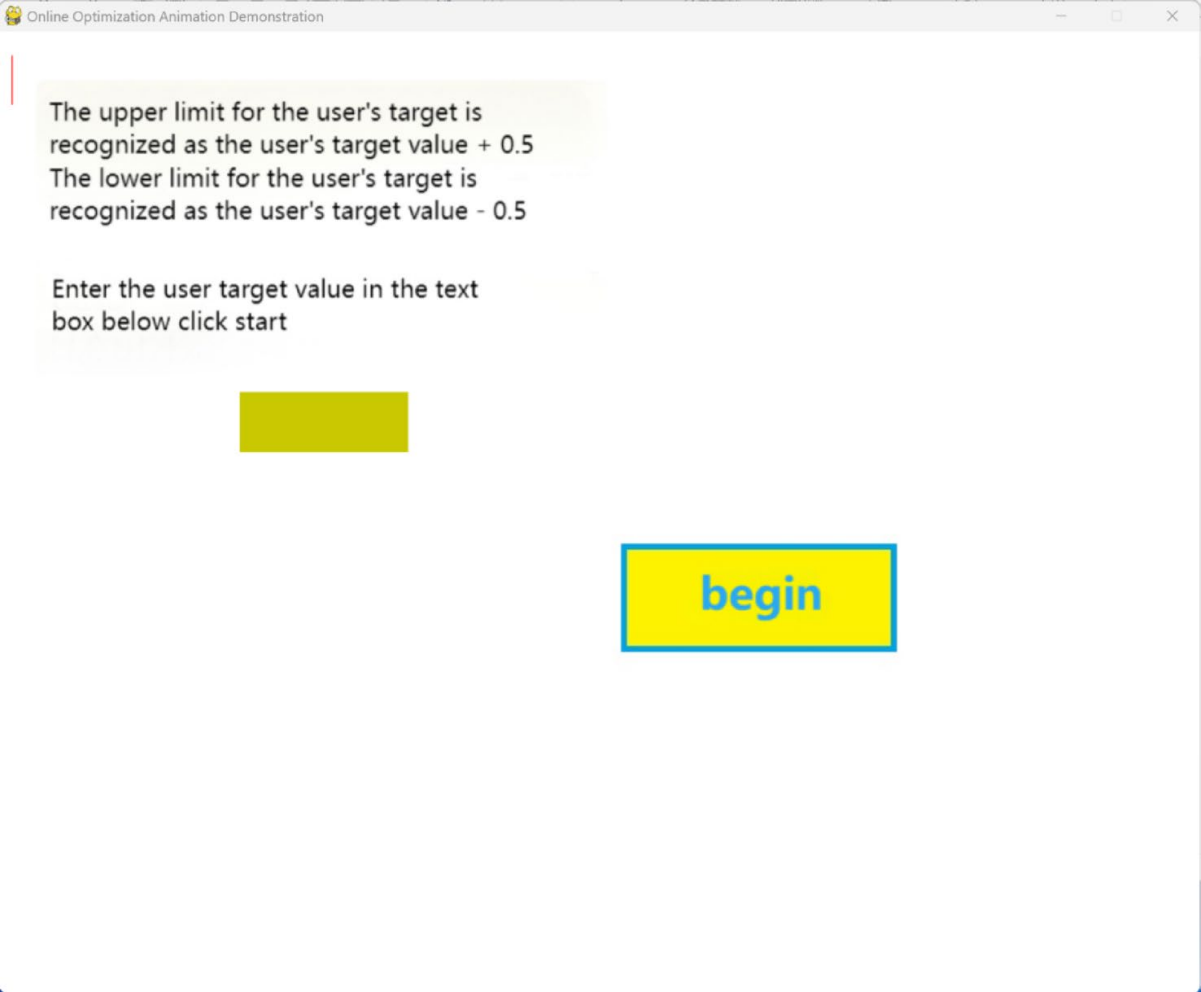
Interactive software input parameter interface.

**Fig. 19:**
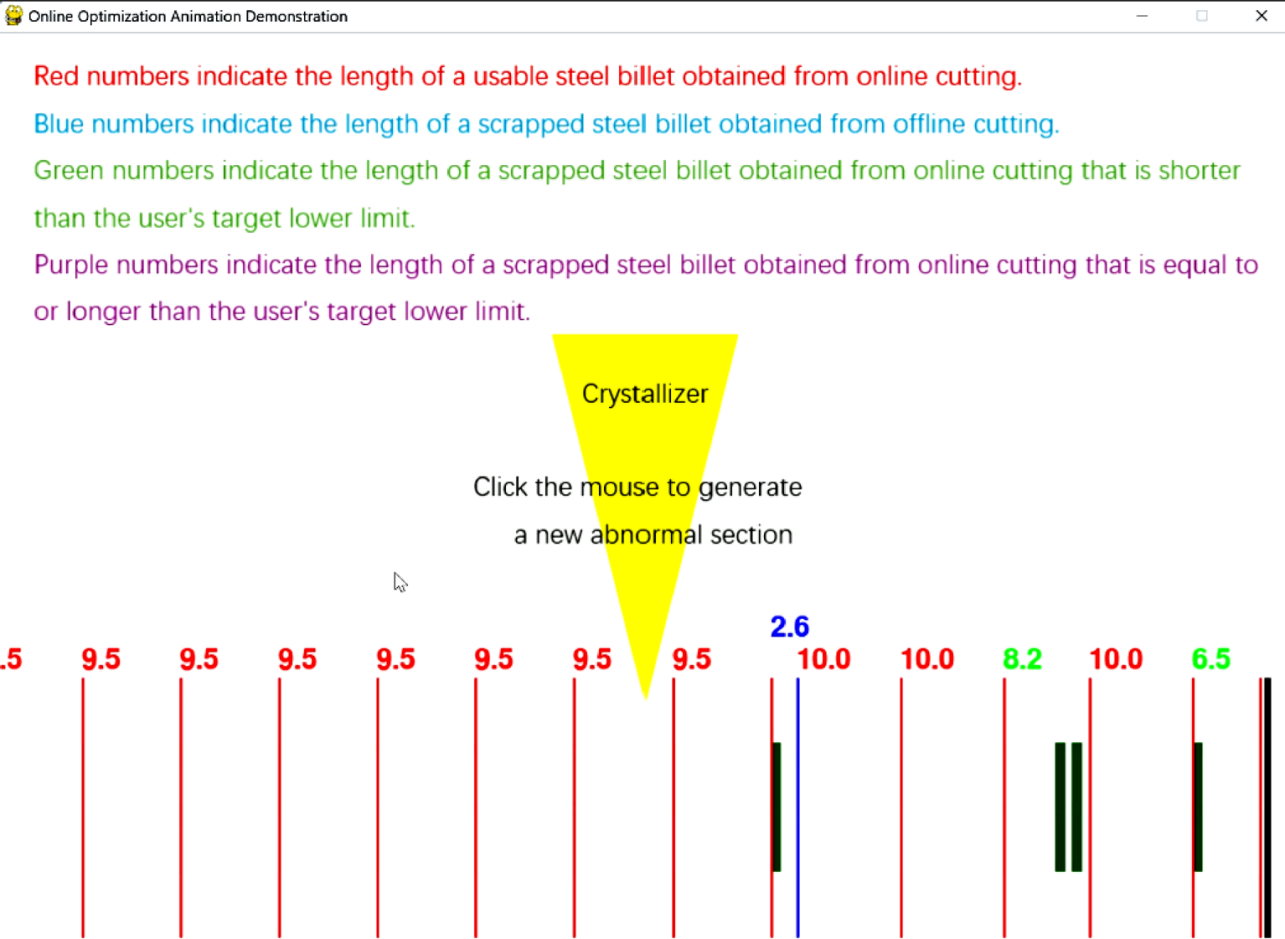
Interactive software animation demonstration interface.

The second one is to input user requirements and then click to start running the animation. During the running process, clicking the left mouse button will immediately generate an abnormal segment in the crystallizer, and the animation will immediately give the real-time optimized subsequent cutting plan, and continue to demonstrate the cutting process in the form of animation. The interface is shown in Figs. 18 and 19.

## Discussion

### Positioning accuracy in practical applications

This paper’s model assumes that abnormal segments occur at integer multiples of 0.1 min. However, in actual production, the occurrence times of abnormal segments often do not meet this precision requirement. In fact, the length of abnormal segments set in the problem is 0.8 m, and in real production, the abnormal part would be contained within these 0.8 m rather than completely filling a uniform 0.8-m shape. Therefore, its abnormal area does not require infinitely high-precision positioning; approximate positioning within a certain error range is sufficient, as long as it ensures that the abnormal part is contained within the positioned abnormal segment. Thus, rounding the occurrence time of abnormal segments to the nearest 0.1-min multiple is reasonable. If higher positioning accuracy is needed in actual production, model parameters can be adjusted accordingly, such as increasing to 0.01 min, though this would increase computation time. Current tests show that 0.1-min positioning accuracy enables rapid model calculation on ordinary computers, far exceeding real-time requirements in actual production. Considering the relatively slow movement of billets in the continuous casting process, the model’s requirements for abnormal segment positioning accuracy and computational capability will not become bottlenecks in applying the algorithm to actual production.

### Applicability and performance discussion of multi-objective optimization techniques

The online optimization algorithm proposed in this paper, by constructing nested models, analytical modeling, and combining it with parameter enumeration, effectively solves the dynamic optimization problem in continuous casting cutting. It significantly improves computational efficiency while ensuring solution quality, meeting the demands of online real-time decision-making. A natural question arises: compared to the specialized optimization path adopted in this paper, could more generalized Multi-objective Optimization Techniques (MOTs)—such as Evolutionary Multi-objective Algorithms (EMOAs)^17,18^, the weighted sum method, or the epsilon-constraint method^19^—bring further performance improvements or offer different solution perspectives for this problem, especially in balancing production speed and efficiency? This section will delve into this issue.

#### Multi-objective handling strategy of the proposed algorithm and its specific advantages

The algorithm proposed in this paper essentially addresses a multi-objective optimization problem, with its core strategy being hierarchical optimization (or lexicographical optimization). Specifically, the optimization objectives are assigned clear priorities:

- *Level 0 Objective (Hard Constraint)*: Ensure the cutting length is between [4.8 m, 12.6 m] to meet basic transportation and production continuity requirements. This directly relates to the stable operation of the production line and can be considered a baseline guarantee for "production speed."
- *Level 1 Objective (Primary Optimization Target)*: Minimize cutting loss. This is core to improving material utilization and directly corresponds to "(material) efficiency."
- *Level 2 Objective*: While satisfying the optimality of the Level 1 objective, maximize the number of steel billets equal to the user’s target length to enhance user satisfaction.
- *Level 3 Objective*: While satisfying the optimality of the first two level objectives, minimize the number of offline secondary cutting operations to reduce the complexity and cost of subsequent processes, indirectly improving "(operational) efficiency."

The advantages of this hierarchical approach are:

- *Clarity of decision-making and industrial relevance*: In industrial practice, the importance of different objectives is often unequal. Hierarchical optimization can directly reflect these priorities, providing a usually unique optimal guidance scheme that aligns with actual production logic.
- *Computational efficiency*: Through analytical modeling, sub-problems at each level (e.g., solving for minimum cutting loss $C\_k(k,L)$ based on a specified k value in Section "[Sec Sec17]") can be solved accurately and quickly. Parameter enumeration is performed within a significantly reduced, effective parameter space derived from problem structure analysis, avoiding a blind search of the original vast solution space. For instance, in static optimization, the traversal range for $k,\;0 \le k \le \left\lfloor {L/Y_{1} } \right\rfloor$, is far smaller than unconstrained cutting combinations. This problem-specific knowledge utilization^20^ is key to the algorithm’s high speed and quality. As indicated in Section "Key innovations and contributions" of this paper, the algorithm’s computational speed far exceeds traditional integer programming methods, owing to a deep understanding and analytical treatment of the problem structure.

#### Characteristics of general multi-objective optimization techniques (MOTs) and potential challenges in this problem

General Multi-objective Optimization Techniques (MOTs) offer a range of methods for handling multiple conflicting objectives^21^. Besides EMOAs, which are population-based heuristic algorithms^22,23^, these include:

- *Scalarization methods*: Such as the Weighted Sum Method, which combines multiple objectives into a single objective function through weighting; and the Epsilon-Constraint Method (ε-constraint method), which optimizes one primary objective while treating others as constraints.
- *Goal programming*: Sets target values for each objective and minimizes deviations from these targets.
- *Heuristic and metaheuristic methods*: Multi-objective versions of algorithms like simulated annealing^24,25^, Genetic Algorithms^26–28^, tabu search^29,30^, as well as EMOAs.

Although these MOTs perform excellently in their respective applicable domains^31^, they generally face the following common challenges when applied to the online continuous casting cutting problem studied in this paper, especially when compared to the specialized algorithm herein:

##### Efficiency of solution space exploration and computational time

- *Scale of the original solution space*: The original solution space for continuous casting cutting (all possible cutting points and length combinations) is combinatorially explosive. Most MOTs (especially heuristic and metaheuristic methods like EMOAs), without substantial guidance from problem-specific prior knowledge, would struggle to find high-quality solutions within a short time in such a vast space.
- *Parameter sensitivity of scalarization methods*: The setting of weights in the weighted sum method significantly impacts the results and is difficult to determine optimally beforehand; similarly, the setting of constraint values in the ε-constraint method is crucial^32^. For dynamically changing online problems, adjusting these parameters in real-time to obtain ideal solutions is very challenging.
- *Computational overhead*: Even EMOAs, designed to explore the Pareto front, their computational model based on "population size × number of generations × individual solution evaluation time" might exceed the seconds-to-milliseconds time window for online decision-making. In contrast, the number of parameter enumerations in this paper’s algorithm (e.g., traversal of *L* in Section "Minimum cutting loss model") is strictly limited and predictable.
- *Potential for "Further Reducing Cutting Loss"*: The Level 1 objective in this paper aims for the mathematical minimum cutting loss through analytical models. If this objective already achieves the theoretical optimum, MOTs cannot “further” reduce it under the same conditions. Their value would lie more in offering trade-off solutions where cutting loss is slightly higher, but other secondary objectives perform significantly better.

##### Precise satisfaction of strict hierarchical optimization objectives

The lexicographical optimization in this paper ensures that the optimality (or feasibility) of higher-priority objectives is absolutely guaranteed before considering lower-priority ones.

The core of most MOTs is to find a “balance” or "trade-off" among different objectives (i.e., the Pareto optimal set)^33^. To make them strictly simulate lexicographical optimization requires special design (e.g., phased optimization, or modifying selection mechanisms in EMOAs), which might weaken their inherent advantages or add complexity. For example, the weighted sum method can simulate some priorities with extreme weights (one weight is 1, others close to 0), but it’s hard to perfectly replicate a multi-level strict hierarchy.

In terms of "ensuring solution quality," the analytical models in this paper can guarantee optimality for sub-problems and specific priorities. MOTs, especially heuristic methods, typically provide approximate solutions and struggle to offer the same level of "guarantee."

##### Perspective on "balancing production speed and efficiency"

Balancing in this Paper’s Method: As mentioned, the Level 0 objective ensures basic production speed, Level 1 and Level 3 objectives focus on material and operational efficiency, respectively, and the algorithm’s fast computation ensures decision-making efficiency.

Balancing via MOTs: MOTs (especially EMOAs) can generate a Pareto front, showcasing various feasible trade-offs among different objectives. For example, decision-makers could see solutions like "sacrificing a small amount of material for faster delivery of specific high-priority orders." This is valuable when decision-makers need more flexible, multi-dimensional alternative solutions and are allowed to deviate from strict lexicographical optimization. However, for online scenarios requiring a quick, single solution compliant with established priorities, this flexibility might translate into decision-making complexity.

#### Limited applicability of MOTs within this problem’s framework

Despite the challenges of directly replacing the specialized algorithm in this paper, MOTs or their underlying ideas might serve as complements in specific contexts:

- *Offline analysis and strategy formulation*: In non-real-time scenarios, MOTs (especially EMOAs) can be used to explore a broader Pareto front, helping to understand potential optimal operating points under different production strategies (e.g., varying user demand priorities, different scrap handling costs), thus providing data support for formulating more macroscopic production plans^34^.
- *Handling non-analytical modules in the model*: If future model extensions involve sub-problems or constraints that are difficult to model analytically, the generality of some MOTs might allow them to serve as solvers for these modules.
- *When decision logic shifts from “single best” to "trade-off among multiple solutions"*: If business requirements permit minor compromises on the highest-priority objective in exchange for significant improvements in other objectives, the diverse set of solutions generated by MOTs would be very useful.

#### Summary

In conclusion, the online optimization algorithm proposed in this paper, based on nested analytical modeling and parameter enumeration, is a solution deeply customized to the characteristics of the continuous casting cutting problem. It achieves a high degree of unity between computational speed and solution quality while strictly adhering to preset optimization priorities, making it highly suitable for online real-time decision-making.

Although general Multi-objective Optimization Techniques (including EMOAs, scalarization methods, etc.) theoretically provide a powerful framework for handling multi-objective conflicts^35^, in this problem, due to the high sensitivity to computation time, the requirement for strict priorities, and the significant efficiency gains already achieved by this paper’s algorithm through problem-specific adaptation, the likelihood of these general MOTs being directly applied to online real-time optimization and outperforming the current algorithm (especially in meeting primary objectives and computational speed) is low. Their greater value may lie in offline strategy analysis, handling non-analytical parts of future model extensions, or providing sets of alternative solutions in decision-making scenarios that allow for more flexible trade-offs. Therefore, under the current problem definition and requirements, the specialized optimization path adopted in this paper is the more appropriate and efficient choice.

### Automation interface between casting equipment and the optimization model

To achieve seamless integration between the continuous‐casting line and the online optimization algorithm—minimizing manual intervention and improving response time—we propose a comprehensive automation interface comprising four key components:

#### Data acquisition and sharing

Leverage the existing SCADA/MES infrastructure or dedicated edge gateways to collect, in real time, critical parameters from the caster (billet position, casting speed, mold temperature, alarm signals, etc.).

Publish these data via industrial protocols (OPC UA, Modbus TCP/IP or EtherNet/IP) into a shared industrial database or message bus (e.g. Apache Kafka, MQTT).

Mirror the cutting machine’s PLC/DCS status and last‐cut position to the same platform, ensuring the optimization model has a global, up-to-date view of the process.

#### Model invocation and online computation

Host the online optimization service on an edge server or industrial cloud, exposing it as a RESTful API or gRPC microservice.

Upon data updates or alarm events, trigger the optimization service to compute and return the optimal cut lengths, cutting sequence, and estimated execution times.

Employ a message-queue or stream-processing framework to enable asynchronous requests and parallel computation, so that even if multiple casters raise alarms simultaneously, the model can respond within milliseconds to seconds.

#### Cutting command dispatch and execution

Once the optimization model issues a cutting plan, use PLC remote‐function calls (RFC) or control‐system scripting to automatically transmit the cut parameters (positions and timing) to the cutting machine controller.

The controller then actuates the cutting valves and blades as specified. Upon completion, the PLC returns execution feedback—cut success/failure and actual deviations—to the data platform, closing the loop for real‐time validation and model recalibration.

#### Safety and fault‐tolerance mechanisms

Implement heartbeat monitoring and dual‐node (hot‐standby) failover: if the edge server or network link fails, the system automatically reverts to a locally stored safe cutting plan, ensuring uninterrupted production.

Apply CRC checks and timestamp synchronization on all critical messages (position, speed, alarms) to prevent errors or latency from causing incorrect cuts.

By deploying this automation interface, the online optimization algorithm and the continuous‐casting/cutting equipment achieve reliable, bidirectional, real-time data exchange and command execution. This eliminates manual data entry and ad-hoc adjustments, dramatically raising the level of automation and fault responsiveness—laying a solid foundation for a truly smart steel mill.

### Practical application potential

Our model not only demonstrates excellent performance in static and online optimization but also possesses high flexibility and scalability to adapt to different production requirements and abnormal situations. Specifically:

1. *Parameter Adjustment*: Through adjusting key parameters in the model (such as cutting length limits, frequency and position of abnormal segments), the model can adapt to different production requirements and abnormal situations. For example, if the billet speed on the production line changes or mold specifications differ, optimal cutting schemes can be regenerated by simply adjusting corresponding model parameters.
2. *Multi-objective Optimization*: The model not only optimizes cutting losses but also considers user satisfaction and offline cutting frequency, allowing flexible adjustment under different priority requirements. Additionally, if new optimization objectives emerge in the future, the model structure facilitates integration of new optimization levels.
3. *Modular Design*: The model adopts a modular design with nested multi-layer optimization and analytical modeling, making it easy to maintain and expand. If new algorithms or optimization strategies need to be introduced, they can be integrated on the existing framework without comprehensive reconstruction.
4. *Software Tool Support*: Through developing automated calculation and dynamic demonstration software, the model’s application scope extends beyond theoretical research to various scenarios in actual production environments. These software tools serve as verification and demonstration tools, proving the algorithm’s feasibility and effectiveness in actual production environments.

### Future research prospects

Although this research is based on a competition problem, its core algorithm’s potential for practical industrial application cannot be ignored. As a complex multi-objective optimization problem in the steel industry, continuous casting cutting can benefit from our proposed online optimization algorithm, which ensures production continuity while reducing material waste and optimizing cutting schemes. Compared to existing manual intervention or semi-automated methods, our automated optimization solution significantly improves calculation speed and accuracy, with strong adaptability and robustness to handle frequent abnormal situations in production processes.

However, actual industrial production demands are more complex than the competition problem, and future research can incorporate more practical production parameters, such as equipment operating status and billet physical properties, to optimize and expand the model. This will help improve the model’s application effectiveness in actual production environments and promote more efficient and intelligent development in the steel manufacturing industry.

## Conclusion

This paper proposes a real-time automated optimization algorithm for continuous casting cutting optimization in the steel industry. Through combining nested models, analytical modeling, and computer traversal algorithms, our proposed solution can effectively handle cutting scheme optimization in static environments and adjust cutting schemes in real-time when abnormalities occur during production, ensuring production continuity and efficiency.

Research results show that this model performs excellently in reducing cutting losses, meeting customer demands, and minimizing secondary cutting operations, particularly in handling various abnormal situations automatically in dynamic production environments. Through further optimization and expansion, this model has potential for widespread application in future intelligent manufacturing and industrial automation.

Overall, this research not only demonstrates academic innovation but also provides new solutions for cutting optimization problems in steel production, offering important reference for future research and applications in related fields.

## Electronic supplementary material

Below is the link to the electronic supplementary material.

Supplementary Material 1

## Data Availability

The software supporting the findings of this study was developed by the authors to dynamically demonstrate and verify the proposed algorithm. It has been deposited in Figshare and is publicly available at 10.6084/m9.figshare.27854517.

## References

[1] Hnatushenko, V. V., Zheldak, T. A. & Koriashkina, L. S. Mathematical model of steel consumption minimization considering the two-stage billets cutting. *Sci. Bull. Natl. Min. Univ.* (2):118–122 (2021). 10.33271/nvngu/2021-2/118

[2] Kong, Y. W., Feng, K., Wang, S. G., Cao, J. F. & Han, Z. W. Metallurgical consideration about abnormal events during slab continuous casting process in the quality diagnosis model. *Appl. Mech. Mater.* **395**, 1179–1183. 10.4028/www.scientific.net/AMM.395-396.1179 (2013).

[3] Sun, L. & Wang, X. Application of mix optimization scheduling approach for steelmaking-continuous casting process based on actual steelmaking industry. *J. Iron. Steel Res. Int.* **20**(10), 1–9. 10.1016/S1006-706X(13)60168-5 (2013).

[4] Wang, D., Xing, F., Kong, X. & Ji, J. Single-objective linear optimization evaluation of used continuous casting and cutting. In *Ninth international conference on mechanical engineering, materials, and automation technology (MMEAT 2023)*, vol. 12801, pp. 1249–1252. (SPIE, 2023), 10.1117/12.3007066

[5] Hu, L. Research on online optimization of continuous casting cutting based on multi-objective linear programming model. *Int. Core J. Eng.* **9**(8), 135–142. 10.6919/ICJE.202308_9(8).0019 (2023).

[6] 2021 Higher education press cup national college student mathematical modeling contest problems [EB/OL], 2021-09-09 [2022-02-05]. Available at: https://www.mcm.edu.cn/html_cn/node/90d223833c1eb50f899aa096a66c6896.html (in Chinese).

[7] Xue, Y. Analysis of the online optimization problem of continuous casting cutting. *Math. Model. Appl.* **11**(1), 82–90. 10.19943/j.2095-3070.jmmia.2022.01.09 (2022) (**in Chinese**).

[8] Cai, Z. J. Online optimization of continuous casting cutting. *Math. Model. Appl.* **11**(2), 67–79. 10.19943/j.2095-3070.jmmia.2022.02.09 (2022) (**in Chinese**).

[9] Evins, R. Multi-level optimization of building design, energy system sizing and operation. *Energy* **90**, 1775–1789. 10.1016/j.energy.2015.07.007 (2015).

[10] Fathy, H. K., Papalambros, P. Y., Ulsoy, A. G., & Hrovat, D. Nested plant/controller optimization with application to combined passive/active automotive suspensions. In *Proceedings of the 2003 American control conference, 2003*, Vol. 4, pp. 3375–3380. (IEEE, 2003).10.1109/ACC.2003.1244053

[11] Hara, S. & Ishihata, M. Approximate and exact enumeration of rule models. In *Proceedings of the AAAI conference on artificial intelligence*, Vol. 32, No. 1. (2018). 10.1609/aaai.v32i1.11637

[12] Creignou, N., Meier, A., Müller, J. S., Schmidt, J. & Vollmer, H. Paradigms for parameterized enumeration. *Theory Comput. Syst.* **60**, 737–758. 10.1007/s00224-016-9702-4 (2017).

[13] Zimmermann, A., Maschotta, R., Wichmann, A. & Hilbrich, R. Optimization of systems with nested design space. In *2018 annual IEEE international systems conference (SysCon)*, pp. 1–8, (IEEE, 2018). 10.1109/SYSCON.2018.8369550

[14] Dörterler, M., Atila, Ü., Top, N. & Şahin, İ. A nested optimization approach for robot gripper multi-objective optimization problem. *Expert Syst. Appl.* **239**, 122163. 10.1016/j.eswa.2023.122163 (2024).

[15] Groppen, V. O. & Berko, A. A. Modular enumeration algorithms: Analytical study and experimental verification of effectiveness. In *Proceedings of SAI intelligent systems conference*, pp. 769–779. (Cham: Springer International Publishing, 2022). 10.1007/978-3-031-16078-3_53

[16] Gergel, V., Grishagin, V. & Israfilov, R. Local tuning in nested scheme of global optimization. *Proc. Comput. Sci.* **51**, 865–874. 10.1016/j.procs.2015.05.216 (2015).

[17] Deb, K. *Multi-objective optimization using evolutionary algorithms* (John Wiley & Sons, 2001).

[18] Li, B., Li, J., Tang, K. & Yao, X. Many-objective evolutionary algorithms: A survey. *ACM Comput. Surv. (CSUR)* **48**(1), 1–35. 10.1145/2792984 (2015).

[19] Bechikh, S., Kessentini, M. & Ben Said, L. Preference incorporation in evolutionary multiobjective optimization: A survey of the state-of-the-art. *Adv. Comput.* **98**, 141–217. 10.1016/bs.adcom.2015.03.001 (2015).

[20] Wolpert, D. H. & Macready, W. G. No free lunch theorems for optimization. *IEEE Trans. Evol. Comput.* **1**(1), 67–82. 10.1109/4235.585893 (1997).

[21] Hwang, C. L. & Masud, A. S. M. *Multiple objective decision making—methods and applications: A state-of-the-art survey* (Springer-Verlag, 1979).

[22] Deb, K., Pratap, A., Agarwal, S. & Meyarivan, T. A fast and elitist multiobjective genetic algorithm: NSGA-II. *IEEE Trans. Evol. Comput.* **6**(2), 182–197. 10.1109/4235.996017 (2002).

[23] Tian, Y., Cheng, R., Zhang, X. & Jin, Y. PlatEMO: A MATLAB platform for evolutionary multi-objective optimization [educational forum]. *IEEE Comput. Intell. Mag.* **12**(4), 73–87. 10.1109/MCI.2017.2742868 (2017).

[24] Guilmeau, T., Chouzenoux, E. & Elvira, V. Simulated annealing: A review and a new scheme. In *2021 IEEE statistical signal processing workshop (SSP)*, pp. 101–105. 10.1109/SSP49050.2021.9513782

[25] Fontes, D. B., Homayouni, S. M. & Goncalves, J. F. A hybrid particle swarm optimization and simulated annealing algorithm for the job shop scheduling problem with transport resources. *Eur. J. Operat. Res.* **306**(3), 1140–1157. 10.1016/j.ejor.2022.09.006 (2023).

[26] Li, W., Tang, R., Wang, X., Zhang, X., Ren, D., Jiang, H. & Wen, Z. A novel approach for computation offloading based on a parallel collaborative genetic algorithm in MEC. *Wireless Personal Commun.*, 1–28 (2025). 10.1007/s11277-025-11760-0

[27] Li, Z. & Zhu, Q. Genetic algorithm-based optimization of offloading and resource allocation in mobile-edge computing. *Information* **11**(2), 83. 10.3390/info11020083 (2020).

[28] Al-Terkawi, L. & Migliavacca, M. An automated parallel genetic algorithm with parametric adaptation for distributed data analysis. *Sci. Rep.* **15**(1), 10836. 10.1038/s41598-025-93943-0 (2025). 40155659 10.1038/s41598-025-93943-0 PMC11953222

[29] Glover, F. & Laguna, M. *Tabu search* 2093–2229 (Springer, 1998).

[30] Prajapati, V. K., Jain, M. & Chouhan, L. Tabu search algorithm (TSA): A comprehensive survey. In *2020 3rd international conference on emerging technologies in computer engineering: Machine learning and internet of things (ICETCE)*, pp. 1–8. (IEEE, 2020). 10.1109/ICETCE48199.2020.9091743

[31] Marler, R. T. & Arora, J. S. Survey of multi-objective optimization methods for engineering. *Struct. Multidiscip. Optim.* **26**(6), 369–395. 10.1007/s00158-003-0368-6 (2004).

[32] Das, I. & Dennis, J. E. A closer look at drawbacks of minimizing weighted sums of objectives for Pareto set generation in multicriteria optimization problems. *Struct. Optim.* **14**(1), 63–69. 10.1007/BF01197559 (1997).

[33] Pareto, V. Manuale di economia politica. Societa Editrice Libraria (1906).

[34] Figueira, J., Greco, S. & Ehrgott, M. *Multiple criteria decision analysis: State of the art surveys* (Springer, 2005).

[35] Ehrgott, M. *Multicriteria optimization* 2nd edn. (Springer, 2005).

