# Correction: Online optimization of continuous casting cutting

**DOI:** 10.1038/s41598-025-17362-x

**Published:** 2025-08-28

**Authors:** Jing Li, Jianjing Zhang, Xiaoru Xing, Sixiao Hu, Yixuan Shi

**Affiliations:** Hebei Institute of Mechanical and Electrical Technology, Xingtai, 054000 Hebei China

Correction to: *Scientific Reports* 10.1038/s41598-025-08908-0, published online 04 July 2025

In the original PDF version of this Article, four equations did not display correctly in the Model establishment and solution section, under the subheading ‘Mathematical expression of existing research limitations’. Consequently,

“As shown in Fig. 3, ${S}_{i}$ represents the coordinate interval between two abnormal segments, and IEq not found represents the sub-interval.”

now reads:

“As shown in Fig. 3, ${S}_{i}$ represents the coordinate interval between two abnormal segments, and ${L}_{i}$ represents the sub-interval.”

“From the perspective of meeting transportation conditions, when at least one interval IEq not found is less than 4.8 m, existing solutions cannot automatically find a solution that satisfies the transportation conditions.”

now reads:

“From the perspective of meeting transportation conditions, when at least one interval ${S}_{i}$ is less than 4.8 m, existing solutions cannot automatically find a solution that satisfies the transportation conditions.”

“From the perspective of minimizing cutting loss, we set the user’s target lower limit as IEq not found.”

now reads:

“From the perspective of minimizing cutting loss, we set the user’s target lower limit as ${Y}_{1}$.”

“For example: If the user’s target lower limit IEq not found meters, and there exists ${S}_{i}=13.7$ meters, dividing ${L}_{i}$ according to ${L}_{i}{=S}_{i}=13.7$ meters, the cutting loss of the ${L}_{i}$ interval will reach 13.7 m.”

now reads:

“For example: If the user’s target lower limit ${Y}_{1}=9.0$ meters, and there exists ${S}_{i}=13.7$ meters, dividing ${L}_{i}$ according to ${L}_{i}{=S}_{i}=13.7$ meters, the cutting loss of the ${L}_{i}$ interval will reach 13.7 m.”

The PDF version of the original Article has been corrected.

