# Supplementary material for: Online optimization of continuous casting cutting
Source: Sci Rep. 2025 Jul 4;15:23942. doi: 10.1038/s41598-025-08908-0 (PMC12227587; doi:10.1038/s41598-025-08908-0)
Supplement: Supplementary file 1 — Supplementary Material 1 [file 41598_2025_8908_MOESM1_ESM.docx]

**Appendix**

**Proof of Sub-case 2.1 in Section 2.3**

Sub-case 2.1 is specifically divided into three sub-cases. Although the schemes for the three sub-cases are the same, each has a different proof process.

**Sub-case 2.1.1**

The billet tail model cutting scheme of $l$, the selected final $k$ value and cutting scheme correspond to Case 1.1 in Section 2.2.1

Proof as follows:

At this point, the total length of utilized (non-cutting loss) steel billets in $L$ has reached the upper limit $l$, cutting loss cannot be further reduced, and it has been optimally divided according to the billet tail model, so it has already achieved the optimal first and second level optimization objectives. There is no offline cutting, so the number of offline cuttings is already minimized.

**Sub-case 2.1.2**

The billet tail model cutting scheme of l, the selected final k value and cutting scheme correspond to Case 1.2 in Section 2.2.1

We use proof by contradiction to prove that the cutting loss is minimized:

In our scheme, the total length of utilized steel billets in $L$ is $kY_{2}$, obviously, $l-kY_{2}\leq k(12.6-Y_{2})$. Suppose there exists a new cutting scheme in L with less cutting loss, in the new scheme, $L_{x}(>kY_{2})$ of steel billets can be utilized. At this point, because $L_{x}>kY_{2}$, the $L_{x}$ part must be $k_{x}(>k)$ steel billets, and the cutting loss of the $l$ part is $l{-L}_{x}$.

Obviously, $l{-L}_{x}<l-kY_{2}\leq k(12.6-Y_{2})<k_{x}(12.6-Y_{2})$, because $l{-L}_{x}<k_{x}(12.6-Y_{2})$, so for l alone (not considering $\Delta L$) there must exist a cutting scheme with cutting loss $l{-L}_{x}$, specifically corresponding to Case 1.2 in Section 2.2.1. This contradicts that the minimum cutting loss of l alone is $l-kY_{2}$, so the assumption does not hold, proving the necessity.

And because it has been optimally divided according to the billet tail model, it has already achieved the optimal first and second level optimization objectives.

According to the billet tail model, it is easy to know that the cutting loss of the l part is less than 4.8. According to the upper limit of $\Delta L$ length in Case 2, the cutting loss of the $L$ part must be less than 12.6. If the cutting loss of the $L$ part is greater than or equal to 4.8, then after step (4), the cutting loss part will be combined into 1 steel billet, so it can be known that the number of offline cuttings is already minimized.

**Sub-case 2.1.3**

The billet tail model cutting scheme of $l$, the selected final $k$ value and cutting scheme correspond to Case 2.2 in Section 2.2.1

Proof as follows:

First, prove that in this sub-case, cutting $l$ according to the billet tail model cannot result in 2 individually scrapped steel billets. The prerequisite for obtaining 2 individually scrapped steel billets is $l\in(12.6K,{KY}_{1}+4.8),K<\frac{4.8}{12.6-Y_{1}},K\in Z^{+}$. At this point, $l$ cannot obtain $K$ usable steel billets, it can only obtain $K-1$ usable steel billets and 2 individually scrapped steel billets. For all user target requirements $Y_{0}\in[8.5,11.1]$, when $Y_{0}\leq10.2$, the upper limit of K is 1, when $Y_{0}\geq10.3$, the upper limit of $K$ is 2. Since Case 2 requires at least 1 usable steel billet, when $K\leq1$, it obviously does not conform to Case 2. We only need to consider the case of $Y_{0}\in[10.3,11.1]$, $K=2$. At this point, cutting l according to the billet tail model will result in $Y_{2}$, $l-Y_{2}-4.8$, $4.8$, three steel billets. At this point, $0.8\leq\Delta L\leq12.6-Y_{2}$, we can get

$$2\times12.6+\Delta L<l+\Delta L=L<2Y_{1}+4.8+12.6-Y_{2}$$

And according to $Y_{0}\in[10.3,11.1]$, we can get

$$2Y_{2}<l<L<3Y_{1}$$

Obviously, cutting $L$ according to the billet tail model (Case 1 scheme), the cutting loss is at least $L-2Y_{2}$. If the loss part is one or two individually scrapped steel billets, it naturally includes the $\Delta L$ part, indicating that this situation belongs to Case 1, and therefore does not belong to Case 2.

If cutting $L$ according to the billet tail model (Case 1 scheme), the cutting loss part is the offline cutting part and only one steel billet undergoes offline cutting, since this part has been adjusted to the end according to the cutting scheme, it belongs to Case 1 by the same reasoning, not Case 2.

If cutting $L$ according to the billet tail model (Case 1 scheme), the cutting loss part is the offline cutting part and two steel billets undergo offline cutting, according to the billet tail model, the cutting loss must be greater than ${12.6-Y}_{2}$ and one of them has an offline cutting part length of ${12.6-Y}_{2}$. According to the Case 1 scheme, this steel billet will be adjusted to the end, obviously $\Delta L$ will also be naturally included in the scrapped part, indicating that this situation belongs to Case 1, not Case 2.

So far, it has been proven that in this sub-case, cutting $l$ according to the billet tail model cannot result in 2 individually scrapped steel billets. That is, cutting $l$ according to the billet tail model results in $k$ usable steel billets with length $Y_{2}$ and one individually scrapped steel billet with length less than $(k+1)Y_{1}{-kY}_{2}$. At this point, the usable part of the entire $L$ can no longer be greater than $kY_{2}$, the cutting loss is already minimized, and since the $l$ part is optimally cut according to the billet tail model, the number of steel billets equal to the user's target value is already maximized, and there is no offline cutting, so the number of offline cuttings is already minimized.
